# Anthropometric measures and adverse outcomes in heart failure with reduced ejection fraction: revisiting the obesity paradox

**DOI:** 10.1093/eurheartj/ehad083

**Published:** 2023-03-22

**Authors:** Jawad H Butt, Mark C Petrie, Pardeep S Jhund, Naveed Sattar, Akshay S Desai, Lars Køber, Jean L Rouleau, Karl Swedberg, Michael R Zile, Scott D Solomon, Milton Packer, John J V McMurray

**Affiliations:** British Heart Foundation Cardiovascular Research Centre, University of Glasgow, Glasgow, UK; Department of Cardiology, Copenhagen University Hospital—Rigshospitalet, Copenhagen, Denmark; British Heart Foundation Cardiovascular Research Centre, University of Glasgow, Glasgow, UK; British Heart Foundation Cardiovascular Research Centre, University of Glasgow, Glasgow, UK; British Heart Foundation Cardiovascular Research Centre, University of Glasgow, Glasgow, UK; Division of Cardiovascular Medicine, Brigham and Women’s Hospital, Boston, MA, USA; Department of Cardiology, Copenhagen University Hospital—Rigshospitalet, Copenhagen, Denmark; Institut de Cardiologie de Montréal, Université de Montréal, Montréal, QC, Canada; Department of Molecular and Clinical Medicine, University of Gothenburg, Gothenburg, Sweden; Department of Medicine, Medical University of South Carolina and Ralph H. Johnson Veterans Administration Medical Center, Charleston, South Carolina, USA; Division of Cardiovascular Medicine, Brigham and Women’s Hospital, Boston, MA, USA; Baylor Heart and Vascular Institute, Baylor University Medical Center, Dallas, TX, USA; British Heart Foundation Cardiovascular Research Centre, University of Glasgow, Glasgow, UK

**Keywords:** Heart failure with reduced ejection fraction, Obesity, Body mass index, Angiotensin receptor-neprilysin inhibitor, Clinical trial

## Abstract

**Aims:**

Although body mass index (BMI) is the most commonly used anthropometric measure, newer indices such as the waist-to-height ratio, better reflect the location and amount of ectopic fat, as well as the weight of the skeleton, and may be more useful.

**Methods and results:**

The prognostic value of several newer anthropometric indices was compared with that of BMI in patients with heart failure (HF) and reduced ejection fraction (HFrEF) enrolled in prospective comparison of ARNI with ACEI to determine impact on global mortality and morbidity in heart failure. The primary outcome was HF hospitalization or cardiovascular death. The association between anthropometric indices and outcomes were comprehensively adjusted for other prognostic variables, including natriuretic peptides. An ‘obesity-survival paradox’ related to lower mortality risk in those with BMI ≥25 kg/m^2^ (compared with normal weight) was identified but this was eliminated by adjustment for other prognostic variables. This paradox was less evident for waist-to-height ratio (as an exemplar of indices not incorporating weight) and eliminated by adjustment: the adjusted hazard ratio (aHR) for all-cause mortality, for quintile 5 vs. quintile 1, was 1.10 [95% confidence interval (CI) 0.87–1.39]. However, both BMI and waist-to-height ratio showed that greater adiposity was associated with a higher risk of the primary outcome and HF hospitalization; this was more evident for waist-to-height ratio and persisted after adjustment e.g. the aHR for HF hospitalization for quintile 5 vs. quintile 1 of waist-to-height ratio was 1.39 (95% CI 1.06–1.81).

**Conclusion:**

In patients with HFrEF, alternative anthropometric measurements showed no evidence for an ‘obesity-survival paradox’. Newer indices that do not incorporate weight showed that greater adiposity was clearly associated with a higher risk of HF hospitalization.

## Structured Graphical Abstract

**Structured Graphical Abstract:**
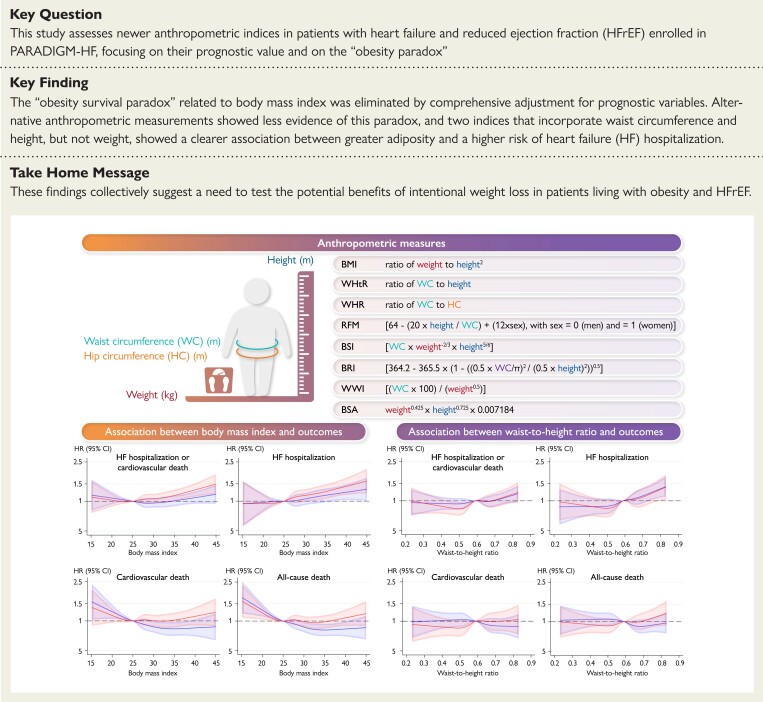
The upper part of the figure describes the calculation of each of the anthropometric measures. The lower part of the figure shows the risk of outcomes according to continuous body mass index (left panel) and waist-to-height ratio (right panel). The solid line represents the hazard ratio and the shaded area the 95% confidence interval. The blue spline is adjusted for treatment and region. The red spline is adjusted for treatment, age, sex, region, systolic blood pressure, heart rate, estimated glomerular filtration rate, left ventricular ejection fraction, log of *n*-terminal pro-B-type natriuretic peptide, body mass index (only in the waist-to-height ratio analyses), New York Heart Association functional class, heart failure aetiology, duration of heart failure, prior heart failure hospitalization, a history of diabetes, and atrial fibrillation. BMI, body mass index; BRI, body roundness index; BSA, body surface area; BSI, body shape index; CI, confidence interval; HF, heart failure; HR, hazard ratio; RFM, relative fat mass; WHR, waist-to-hip ratio; WHtR, waist-to-height ratio; WWI, weight-adjusted-waist index.

**See the editorial comment for this article ‘Revisiting the obesity paradox in heart failure: what is the best anthropometric index to gauge obesity?’, by R. Sato and S. von Haehling, https://doi.org10.1093/eurheartj/ehad079.**

## Introduction

Although obesity has been repeatedly shown to be an independent risk factor for the development of heart failure (HF),^1–3^ its prognostic importance in established HF, especially HF with reduced ejection fraction (HFrEF), is less clear and an ‘obesity-survival paradox’ has been described in patients with HFrEF.^4–8^ However, the associations between obesity and outcomes in HFrEF have generally been based on body mass index (BMI), calculated as weight in kilograms divided as height in meters squared, which has many limitations as a measure of adiposity. BMI does not take into account the location of body fat or its amount, relative to muscle, or the weight of the skeleton, which may differ according to sex, age, and race.^9–12^ In HF specifically, there is also the contribution of retained fluid to body weight. Consequently, alternative anthropometric indices have been proposed such as waist circumference, waist-to-hip ratio, and weight-adjusted-weight index, which may better reflect intra-abdominal fat (‘central obesity’) and body shape index, body roundness index, and relative fat mass, which have been suggested to better reflect the distribution of body fat and total fat mass.^13–17^ Recently, some of these were found to be better predictors of incident HF in the general population than BMI,^18–20^ but they are not commonly measured in clinical practice.

Waist-to-height ratio is of particular interest as it should, to some extent, take account of sex- and race-based differences in stature and the distribution of body fat, and the National Institute for Health and Care Excellence in the United Kingdom has recently suggested that waist-to-height ratio should replace BMI in the evaluation of adiposity.^21,22^

To complicate matters further, the association between BMI (and potentially any other anthropometric index) and outcome in patients with HFrEF is also confounded by the relationship between adiposity and natriuretic peptide levels. Higher BMI is associated with lower natriuretic peptide levels, possibly due to increased clearance or potentially because patients with obesity present with symptoms at an earlier stage in the development of HF.^23,24^ Although natriuretic peptide level is one of the most powerful prognostic variables in HFrEF,^25^ few analyses of the association between BMI (or other anthropometric indices) and outcome have accounted for this.^4–8,26–34^ Therefore, we have examined the newer anthropometric indices described above in patients with HFrEF, focusing on their prognostic value and whether an ‘obesity-survival paradox’ is observed, as has been reported for BMI. We have also adjusted all analyses for natriuretic peptide levels. We carried out these analyses in a global population enrolled in the prospective comparison of angiotensin receptor-neprilysin inhibitor with angiotensin-converting enzyme inhibitor (ACE-i) to determine impact on global mortality and morbidity in heart failure trial (prospective comparison of ARNI with ACEI to determine impact on global mortality and morbidity in heart failure [PARADIGM-HF]) which included 1832 women and 6567 men with HFrEF enrolled in 47 countries on six continents.^35^

## Methods

PARADIGM-HF was a randomized, double-blind, placebo-controlled trial in patients with chronic HFrEF, evaluating the efficacy and safety of the angiotensin receptor-neprilysin inhibitor sacubitril/valsartan compared with enalapril, added to standard care. The design and primary results of PARADIGM-HF have been reported previously.^35,36^ The institutional review boards of all participating institutions approved the protocol, and all patients gave written informed consent.

### Patients and study procedures

Key inclusion criteria were age ≥18 years, New York Heart Association (NYHA) functional class II-IV, left ventricular ejection fraction (LVEF) of ≤35% (changed from ≤40% by a protocol amendment), elevated natriuretic peptide levels, and treatment with a stable dose of an ACE-i or angiotensin receptor blocker (ARB) equivalent to enalapril 10 mg/day for at least 4 weeks before the screening visit. Key exclusion criteria were symptomatic hypotension or systolic blood pressure <95 mmHg at randomization (100 mmHg at screening), estimated glomerular filtration rate (eGFR) < 30 mL/min/1.73 m^2^ at randomization (or screening), potassium >5.4 mmol/L at randomization (>5.2 mmol/L at screening), a history of angioedema, and intolerance to ACE-i or ARB.^36^

On trial entry, ongoing therapy with ACE-i or ARB was stopped, and patients received enalapril 10 mg twice daily for 2 weeks followed by sacubitril/valsartan, up-titrated from 100 mg twice daily to 200 mg twice daily, for additional 4–6 weeks. Patients tolerating both drugs at the target doses were then randomly assigned to double-blind therapy with sacubitril/valsartan or enalapril in a 1:1 ratio.^36^

### Anthropometric measures

**Figure 1:**
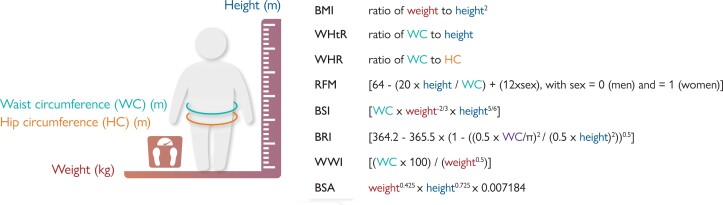
Calculation of anthropometric measures. This figure describes the calculation of each of the anthropometric measures. BMI, body mass index; BRI, body roundness index; BSA, body surface area; BSI, body shape index; RFM, relative fat mass; WHR, waist-to-hip ratio; WHtR, waist-to-height ratio; WWI, weight-adjusted-waist index.

Data on anthropometric measures were obtained at the randomization visit. The calculation of each of these measures is described in *Figure 1*.

In the analyses using BMI, patients were divided according to the World Health Organization (WHO) BMI categories, i.e. underweight (<18.5 kg/m^2^); normal weight (18.5–24.9 kg/m^2^); overweight (25.0–29.9 kg/m^2^); obesity class I (30.0–34.9 kg/m^2^); obesity class II (35.0–39.9 kg/m^2^) and obesity class III (≥40 kg/m^2^). The choice of waist-to-height ratio as the exemplar anthropometric index that does not include weight was prespecified as it is easiest to calculate. We analyzed this index by quintile as the lowest quintile identified patients with a waist-to-height ratio in the range 0.150–0.520, the upper end of which approximates the <0.5 regarded as healthy.

### Outcomes

The primary outcome in PARADIGM-HF was the composite of HF hospitalization or cardiovascular death. In the present analysis, we also examined each of the components of the primary outcome, non-cardiovascular death, and death from any cause.

### Statistical analyses

Baseline characteristics were summarized as frequencies with percentages, means with standard deviation (SD), or medians with interquartile ranges. Differences in baseline characteristics were tested using the Cochran–Armitage trend test for binary variables, the Cochran–Mantel–Haenszel test for categorical variables, and the Jonckheere–Terpstra test and linear regression for non-normal and normally distributed continuous variables, respectively.

Time-to-event data, regardless of treatment allocation, were evaluated using Cox proportional-hazards models, adjusted for treatment-group assignment and geographic region, and hazard ratios (HR) with 95% confidence intervals (CIs) were reported. In addition, HRs adjusted for treatment-group assignment, age, sex, race, geographic region, systolic blood pressure, heart rate, eGFR, LVEF, BMI (not included when analyzing outcomes according to BMI), log of *n*-terminal pro-B-type natriuretic peptide (NT-proBNP), NYHA functional class, HF aetiology, duration of HF, prior HF hospitalization, and a history of diabetes and atrial fibrillation were reported. The relationship between anthropometric measurements as continuous variables and the risk of outcomes was also examined in restricted cubic spline analyses (with the median value as reference).

To compare the effects of sacubitril/valsartan vs. enalapril, time-to-event data were evaluated with Cox proportional-hazards models, with geographical region and treatment-group assignment as fixed-effect factors.

All analyses were conducted using SAS version 9.4 (SAS Institute, Cary, NC) and STATA version 17.0 (College Station, TX). A *P*-value of .05 was considered statistically significant.

## Results

Of the 8399 patients randomized in PARADIGM-HF, data on BMI were available in 99.9% (as were data on body surface area), and data on waist-to-height ratio were available in 98.6% (as were data on waist circumference, relative fat mass, body roundness index, body shape index, and weight-adjusted-weight index although our focus was on waist-to-height ratio). Waist-to-hip ratio was available in 97.7% of randomized patients. Median duration of follow-up was 27 months (25th-75th percentile, 19–36 months).

### Patient characteristics

#### Body mass index

**Figure 2:**
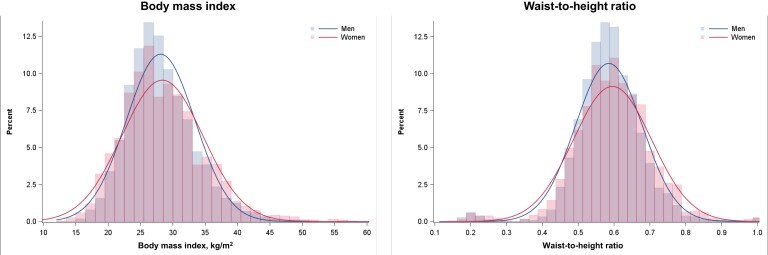
Distribution of body mass index and waist-to-height-ratio according to sex. This figure shows the frequency distribution curves of body mass index and waist-to-height ratio, respectively, according to sex. The red line and bars represent women, and the blue line and bars represent men.

Median BMI was 27.5 kg/m^2^ (25th-75th percentile, 24.5–31.0 kg/m^2^) and 27.6 kg/m^2^ (25th-75th percentile, 24.0–32.0 kg/m^2^) in men and women, respectively (*Figure 2A*). In total, 153 patients had a BMI <18.5 kg/m^2^; 2268 patients between 18.5–24.9 kg/m^2^; 3249 patients between 25–29.9 kg/m^2^; 1810 patients between 30–34.9 kg/m^2^; and 909 patients ≥35 kg/m^2^. Detailed baseline characteristics according to these BMI categories are presented in *Table 1a*. Compared with patients with normal weight, those with higher BMI were younger, more often female, and white. Patients with higher BMI had more comorbidity, longer duration HF, more prior hospitalization for HF, and worse NYHA functional class, and Kansas City Cardiomyopathy Questionnaire (KCCQ) scores. However, they had higher LVEF and were more likely to have a non-ischemic aetiology (although patients with a BMI <18.5 kg/m^2^ were more likely than the other BMI categories to have a non-ischemic aetiology). Patients with higher BMI had lower natriuretic peptide levels (irrespective of the presence of atrial fibrillation) but a higher urinary cGMP/BNP ratio (as a marker of tissue responsiveness to natriuretic peptides). Patients with higher BMI had higher aldosterone, hemoglobin, uric acid, and blood urea nitrogen, levels, as well as neutrophil/lymphocyte ratio, compared with patients with normal weight, but a lower eGFR (*Table 1a*).

**Table 1a:** Baseline characteristics according to body mass index

	Underweight	Normal weight	Overweight	Obesity class I	Obesity class II/III	*P*-value
	BMI <18.5	BMI 18.5–24.9	BMI 25–29.9	BMI 30–34.9	BMI ≥35	
	*n* = 153	*n* = 2268	*n* = 3249	*n* = 1810	*n* = 909	
Age (years), mean (SD)	60.9 ± 13.9	64.5 ± 12.2	64.6 ± 11.1	63.4 ± 10.7	60.3 ± 10.4	<.001
Male sex, *n* (%)	89 (58.2)	1776 (78.3)	2635 (81.1)	1417 (78.3)	643 (70.7)	.04
**Geographic region, *n* (%)**						<.001
ȃNorth America	3 (2.0)	98 (4.3)	208 (6.4)	158 (8.7)	135 (14.9)	
ȃLatin America	24 (15.7)	420 (18.5)	601 (18.5)	295 (16.3)	91 (10.0)	
ȃWestern Europe and other	16 (10.5)	427 (18.8)	875 (26.9)	489 (27.0)	238 (26.2)	
ȃCentral Europe	12 (7.8)	541 (23.9)	1090 (33.5)	755 (41.7)	428 (47.1)	
ȃAsia-Pacific	98 (64.1)	782 (34.5)	475 (14.6)	113 (6.2)	17 (1.9)	
**Race, *n* (%)**						<.001
ȃWhite	30 (19.6)	1092 (48.1)	2238 (68.9)	1419 (78.4)	758 (83.4)	
ȃBlack	8 (5.2)	134 (5.9)	127 (3.9)	83 (4.6)	76 (8.4)	
ȃAsian	99 (64.7)	785 (34.6)	488 (15.0)	115 (6.4)	20 (2.2)	
ȃOther	16 (10.5)	257 (11.3)	396 (12.2)	193 (10.7)	55 (6.1)	
**Physiological measures, median (IQR) or mean (SD)**						
ȃSystolic blood pressure (mmHg)	118.4 ± 15.5	118.7 ± 14.8	121.4 ± 15.0	123.4 ± 15.6	124.7 ± 15.8	<.001
ȃHeart rate (bpm)	76.1 ± 11.3	71.8 ± 11.6	71.8 ± 11.9	72.8 ± 12.4	74.4 ± 12.6	<.001
ȃBody mass index	17.5 (16.9–18.0)	23.0 (21.5–24.1)	27.2 (26.1–28.5)	31.9 (30.8–33.1)	37.7 (36.1–40.4)	<.001
ȃWaist-to-height ratio	0.46 (0.42–0.49)	0.52 (0.48–0.55)	0.58 (0.55–0.61)	0.64 (0.61–0.67)	0.71 (0.67–0.76)	<.001
ȃWaist circumference	75 (68–81)	87 (81–93)	99 (92–104)	109 (103–115)	121 (113–130)	<.001
ȃWaist-to-hip ratio	0.91 (0.84–0.94)	0.93 (0.88–0.98)	0.96 (0.92–1.02)	0.99 (0.94–1.05)	1.00 (0.94–1.06)	<.001
ȃRelative fat mass	23.8 (18.9–31.4)	26.7 (23.6–30.0)	30.3 (28.1–33.1)	33.4 (31.7–36.1)	37.2 (35.0–44.7)	<.001
ȃWeight-adjusted-waist index	11.0 (10.1–12.0)	11.0 (10.3–11.5)	11.1 (10.6–11.7)	11.3 (10.8–11.9)	11.5 (10.9–12.1)	<.001
ȃBody shape index	0.088 (0.080–0.095)	0.084 (0.080–0.088)	0.084 (0.080–0.087)	0.083 (0.079–0.087)	0.082 (0.078–0.086)	<.001
ȃBody roundness index	2.6 (1.9–3.3)	3.8 (3.1–4.4)	5.0 (4.3–5.7)	6.4 (5.7–7.2)	8.2 (7.1–9.5)	<.001
Current smoker, *n* (%)	26 (17.0)	380 (16.8)	455 (14.0)	231 (12.8)	116 (12.8)	<.001
Ischemic cause of HF, *n* (%)	69 (45.1)	1371 (60.4)	2006 (61.7)	1079 (59.6)	504 (55.4)	.22
Duration of HF, *n* (%)						<.001
ȃ<=1 year	87 (56.9)	814 (35.9)	929 (28.6)	475 (26.2)	215 (23.7)	
ȃ1–5 years	53 (34.6)	878 (38.7)	1270 (39.1)	667 (36.9)	361 (39.7)	
ȃ> 5 years	13 (8.5)	576 (25.4)	1050 (32.3)	668 (36.9)	333 (36.6)	
LVEF, mean (SD)	26.5 ± 6.4	28.8 ± 6.3	29.5 ± 6.1	30.0 ± 6.1	30.5 ± 6.1	<.001
**NYHA class at randomization, *n* (%)**						<.001
ȃI	11 (7.2)	130 (5.7)	150 (4.6)	72 (4.0)	25 (2.8)	
ȃII	122 (79.7)	1667 (73.6)	2325 (71.7)	1256 (69.5)	541 (59.6)	
ȃIII	20 (13.1)	455 (20.1)	745 (23.0)	462 (25.6)	335 (36.9)	
ȃIV	0 (0.0)	14 (0.6)	24 (0.7)	16 (0.9)	6 (0.7)	
KCCQ-OSS, mean (SD)	74.7 ± 18.6	75.0 ± 18.5	74.6 ± 18.6	71.2 ± 20.3	65.5 ± 21.1	<.001
KCCQ-CSS, mean (SD)	79.6 ± 17.1	78.9 ± 18.1	77.5 ± 18.4	73.8 ± 20.2	67.8 ± 20.8	<.001
**Medical history, *n* (%)**						
ȃHospitalization for HF	85 (55.6)	1343 (59.2)	2053 (63.2)	1165 (64.4)	623 (68.5)	<.001
ȃHypertension	65 (42.5)	1360 (60.0)	2290 (70.5)	1427 (78.8)	792 (87.1)	<.001
ȃDiabetes	23 (15.0)	579 (25.5)	1083 (33.3)	747 (41.3)	471 (51.8)	<.001
ȃHistory of atrial fibrillation	17 (11.1)	640 (28.2)	1191 (36.7)	779 (43.0)	459 (50.5)	<.001
ȃAtrial fibrillation on electrocardiogram	10 (6.6)	398 (17.9)	784 (24.6)	524 (29.2)	318 (35.5)	<.001
ȃPrevious myocardial infarction	30 (19.6)	934 (41.2)	1525 (46.9)	785 (43.4)	355 (39.1)	.32
ȃPrevious stroke	8 (5.2)	189 (8.3)	279 (8.6)	154 (8.5)	95 (10.5)	.06
ȃChronic obstructive pulmonary disease	22 (14.4)	275 (12.1)	389 (12.0)	240 (13.3)	154 (16.9)	.002
ȃCancer	6 (3.9)	91 (4.0)	175 (5.4)	91 (5.0)	49 (5.4)	.08
**Treatment, *n* (%)**						
ȃBeta-blocker	134 (87.6)	2076 (91.5)	3006 (92.5)	1720 (95.0)	865 (95.2)	<.001
ȃMineralocorticoid-receptor antagonist	84 (54.9)	1244 (54.9)	1786 (55.0)	1030 (56.9)	522 (57.4)	.09
ȃDiuretic	123 (80.4)	1720 (75.8)	2564 (78.9)	1518 (83.9)	804 (88.4)	<.001
ȃDigitalis	62 (40.5)	817 (36.0)	906 (27.9)	476 (26.3)	274 (30.1)	<.001
ȃAntiplatelet	87 (56.9)	1323 (58.3)	1902 (58.5)	961 (53.1)	459 (50.5)	<.001
ȃOral anticoagulant	11 (7.2)	536 (23.6)	1028 (31.6)	706 (39.0)	400 (44.0)	<.001
ȃStatin	67 (43.8)	1142 (50.4)	1907 (58.7)	1079 (59.6)	525 (57.8)	<.001
ȃImplantable cardioverter-defibrillator	5 (3.3)	225 (9.9)	519 (16.0)	329 (18.2)	163 (17.9)	<.001
ȃCardiac resynchronization therapy	1 (0.7)	129 (5.7)	245 (7.5)	122 (6.7)	75 (8.3)	.002
Biomarkers, median (IQR) or mean (SD) ^a^						
**NT-proBNP (pg/mL)**						
ȃNo atrial fibrillation on electrocardiogram	2510 (1225–6807)	1964 (991–4371)	1450 (831–2836)	1229 (728–2196)	1072 (672–1938)	<.001
ȃAtrial fibrillation on electrocardiogram	3515 (1605–10835)	2893 (1632–5715)	2102 (1203–4053)	1748 (1113–3231)	1508 (876–2468)	<.001
**BNP (pg/mL)**						
ȃNo atrial fibrillation on electrocardiogram	428 (159–763)	309 (167–633)	254 (151–448)	223 (141–391)	208 (132–346)	<.001
ȃAtrial fibrillation on electrocardiogram	578 (215–1167)	334 (195–664)	264 (173–471)	221 (146–396)	194 (143–290)	<.001
**ucGMP/BNP ratio (nmol/pg)**						
ȃNo atrial fibrillation on electrocardiogram	1.5 (1.0–3.5)	3.2 (1.6–6.7)	4.6 (2.6–7.9)	4.8 (2.6–7.6)	5.2 (2.7–10.2)	<.001
ȃAtrial fibrillation on electrocardiogram	2.8 (0.9–3.4)	5.1 (2.9–8.4)	5.1 (2.6–10.0)	5.7 (3.6–9.0)	7.5 (4.1–11.1)	.001
**Aldosterone (pmol/L)**						
ȃNo mineralocorticoid-receptor antagonist	246 (179–566)	180 (126–269)	212 (138–289)	221 (136–327)	260 (165–355)	<.001
ȃMineralocorticoid-receptor antagonist	322 (98–430)	262 (155–394)	261 (158–466)	327 (190–535)	325 (196–597)	.001
Troponin T (ng/L)	19 (8–26)	15 (10–24)	15 (10–23)	15 (10–23)	14 (9–22)	.59
ST2 (ng/mL)	22 (18–37)	33 (26–43)	32 (25–41)	33 (27–42)	32 (25–40)	.90
MMP-9 (ng/mL)	119 (53–173)	58 (35–121)	63 (38–119)	64 (41–126)	78 (44–161)	.005
MMP-2 (ng/mL)	147 (133–173)	133 (114–157)	134 (115–154)	135 (118–159)	137 (119–158)	.13
TIMP1 (ng/mL)	158 (137–199)	127 (104–158)	122 (104–149)	123 (105–148)	129 (106–153)	.96
Galectin 3 (ng/mL)	24 (21–30)	17 (14–23)	17 (14–21)	17 (14–21)	18 (15–22)	.11
GDF-15 (ng/L)	2238 (1983–4798)	1805 (1176–2536)	1590 (1153–2298)	1695 (1219–2466)	1598 (1074–2404)	.20
Hemoglobin (g/L)	128 (119–139)	135 (125–146)	140 (130–151)	143 (132–153)	143 (132–154)	<.001
Hemoglobin A1c (%)	6.0 (5.7–6.4)	6.1 (5.7–6.6)	6.2 (5.8–6.8)	6.3 (5.9–7.0)	6.5 (6.0–7.5)	<.001
White blood cell count (10^9^/L)	6.4 (5.6–8.1)	6.5 (5.4–7.8)	6.7 (5.7–8.0)	6.8 (5.8–8.1)	7.2 (6.0–8.6)	<.001
Neutrophil count (10^9^/L)	3.9 (3.1–5.1)	4.0 (3.1–5.0)	4.2 (3.3–5.2)	4.3 (3.5–5.3)	4.5 (3.6–5.6)	<.001
Neutrophil/lymphocyte ratio	2.1 (1.6–3.1)	2.3 (1.7–3.1)	2.4 (1.7–3.2)	2.4 (1.8–3.2)	2.4 (1.8–3.4)	<.001
Monocyte count (10^9^/L)	0.4 (0.3–0.5)	0.4 (0.3–0.6)	0.5 (0.4–0.6)	0.5 (0.4–0.6)	0.5 (0.4–0.6)	<.001
Lymphocyte count (10^9^/L)	1.8 (1.4–2.2)	1.7 (1.3–2.2)	1.8 (1.4–2.2)	1.8 (1.4–2.2)	1.8 (1.5–2.3)	<.001
eGFR (mL/min/1.73 m^2^)	69 (56–86)	68 (55–82)	66 (54–79)	65 (53–78)	66 (53–79)	<.001
Creatinine (*μ*mol/L)	87 (74–101)	93 (80–111)	96 (82–114)	96 (82–116)	96 (80–114)	<.001
Blood urea nitrogen (mmol/L)	5.4 (3.9–8.0)	6.4 (5.0–8.6)	6.8 (5.4–8.9)	6.8 (5.7–8.6)	6.8 (5.4–8.9)	<.001
Cystatin (mg/L)	1.4 (1.2–1.7)	1.2 (1.0–1.5)	1.2 (1.0–1.4)	1.1 (1.0–1.4)	1.2 (1.0–1.4)	.40
**Urine albumin-creatinine ratio (mg/mmol)**						
ȃNo diabetes	3.9 (0.9–8.1)	0.8 (0.2–2.3)	0.9 (0.3–2.6)	0.8 (0.2–2.8)	1.1 (0.3–3.2)	.22
ȃDiabetes	4.3 (2.7–5.8)	1.5 (0.5–6.1)	1.7 (0.6–7.1)	2.1 (0.7–6.3)	1.9 (0.7–8.1)	.17
KIM-1 (pg/mL)	260 (165–318)	124 (84–185)	120 (81–179)	134 (92–201)	149 (99–220)	<.001
Uric acid (*μ*mol/L)	357 (297–434)	381 (321–464)	399 (327–482)	404 (339–488)	428 (351–512)	<.001
ALT (IU/L)	16 (11–21)	17 (13–22)	18 (14–24)	19 (14–25)	19 (15–25)	<.001
AST (IU/L)	23 (19–27)	21 (18–26)	21 (17–25)	20 (17–25)	20 (17–24)	<.001
Alkaline phosphatase (IU/L)	79 (65–97)	74 (60–92)	71 (58–88)	70 (57–87)	71 (58–88)	<.001
Total bilirubin (*μ*mol/L)	9 (7–14)	9 (7–14)	9 (7–14)	9 (7–12)	9 (7–12)	<.001
Albumin (g/L)	42 (39–44)	43 (41–45)	43 (41–45)	43 (41–45)	42 (40–44)	.44

BNP, B-type natriuretic peptide; CSS, clinical summary score; eGFR, estimated glomerular filtration rate; HF, heart failure; KCCQ, Kansas City Cardiomyopathy Questionnaire; LVEF, left ventricular ejection fraction; NYHA, New York Heart Association; NT-proBNP, *n*-terminal pro-B-type natriuretic peptide; OSS, overall summary score; SD, standard deviation; ucGMP, urinary cyclic guanosine monophosphate.

Randomization visit, except ST2, MMP2, MMP9, TIMP1, GDF-15, and KIM-1, which were collected at the screening visit. Number of patients with missing biomarkers are: NT-proBNP, *n* = 14; BNP, *n* = 59; ucGMP/BNP ratio, *n* = 6375; aldosterone, *n* = 6381; troponin T, *n* = 6345; ST2, *n* = 6803; MMP-9, *n* = 6999; MMP-2, *n* = 6998; TIMP1, *n* = 6983; galectin 3, *n* = 6354; GDF-15, *n* = 6850; Hemoglobin, *n* = 274; hemoglobin A1c, *n* = 180; white blood cell count, *n* = 294; neutrophil count, *n* = 421; neutrophil/lymphocyte ratio, *n* = 421; monocyte count, *n* = 421; lymphocyte count, *n* = 421; eGFR, *n* = 0; creatinine, *n* = 0; blood urea nitrogen, *n* = 161; Cystatin, *n* = 6343; urine albumin-creatinine ratio, *n* = 6416; KIM-1, *n* = 6846; uric acid, *n* = 186; ALT, *n* = 219; AST, *n* = 236; alkaline phosphatase, *n* = 199; total bilirubin, *n* = 199; albumin, *n* = 179.

#### Waist-to-height ratio

Median waist-to-height ratio was 0.58 (25th-75th percentile, 0.54–0.64) and 0.59 (25th-75th percentile, 0.53–0.66) in men and women, respectively (*Figure 2B*). Baseline characteristics of patients according to quintiles of waist-to-height ratio are presented in *Table 1b*. Overall, the differences were similar to those described above and in *Table 1a*.

**Table 1b:** Baseline characteristics according to quintile of waist-to-height ratio

	Quintile 1	Quintile 2	Quintile 3	Quintile 4	Quintile 5	*P*-value
	*n* = 1635	*n* = 1659	*n* = 1658	*n* = 1655	*n* = 1674	
Age (years), mean (SD)	62.3 ± 12.9	64.1 ± 11.3	64.3 ± 11.2	64.9 ± 10.7	63.4 ± 10.7	.001
Male sex, *n* (%)	1250 (76.5)	1346 (81.1)	1370 (82.6)	1314 (79.4)	1193 (71.3)	<.001
**Geographic region, *n* (%)**						<.001
ȃNorth America	103 (6.3)	94 (5.7)	115 (6.9)	119 (7.2)	161 (9.6)	
ȃLatin America	239 (14.6)	285 (17.2)	304 (18.3)	305 (18.4)	275 (16.4)	
ȃWestern Europe and other	243 (14.9)	386 (23.3)	415 (25.0)	458 (27.7)	479 (28.6)	
ȃCentral Europe	446 (27.3)	507 (30.6)	572 (34.5)	616 (37.2)	679 (40.6)	
ȃAsia-Pacific	604 (36.9)	387 (23.3)	252 (15.2)	157 (9.5)	80 (4.8)	
**Race, *n* (%)**						<.001
ȃWhite	769 (47.0)	1006 (60.6)	1108 (66.8)	1232 (74.4)	1332 (79.6)	
ȃBlack	118 (7.2)	87 (5.2)	64 (3.9)	70 (4.2)	82 (4.9)	
ȃAsian	606 (37.1)	389 (23.4)	261 (15.7)	161 (9.7)	85 (5.1)	
ȃOther	142 (8.7)	177 (10.7)	225 (13.6)	192 (11.6)	175 (10.5)	
**Physiological measures, median (IQR) or mean (SD)**						
ȃSystolic blood pressure (mmHg)	118.7 ± 14.5	119.5 ± 14.8	121.8 ± 15.2	122.5 ± 15.2	124.3 ± 16.0	<.001
ȃHeart rate (bpm)	72.3 ± 11.6	71.8 ± 12.1	71.9 ± 11.6	72.1 ± 12.3	73.8 ± 12.3	<.001
ȃBody mass index	22.7 (20.6–24.7)	25.2 (23.6–26.9)	27.4 (25.6–29.4)	30.0 (27.9–32.1)	34.4 (31.2–37.7)	<.001
ȃWaist-to-height ratio	0.48 (0.46–0.51)	0.55 (0.53–0.56)	0.59 (0.58–0.59)	0.63 (0.62–0.64)	0.70 (0.67–0.74)	<.001
ȃWaist circumference	81 (75–86)	92 (89–96)	99 (96–103)	107 (102–110)	118 (112–125)	<.001
ȃWaist-to-hip ratio	0.90 (0.85–0.95)	0.94 (0.90–0.98)	0.97 (0.93–1.01)	0.99 (0.95–1.04)	1.02 (0.97–1.08)	<.001
ȃRelative fat mass	23.8 (21.1–25.4)	27.7 (26.7–28.5)	30.0 (29.4–30.7)	32.5 (31.7–33.3)	36.4 (34.6–46.0)	<.001
ȃWeight-adjusted-waist index	10.1 (9.6–10.6)	10.8 (10.5–11.2)	11.2 (10.8–11.6)	11.5 (11.1–11.9)	12.0 (11.6–12.5)	<.001
ȃBody shape index	0.078 (0.073–0.083)	0.083 (0.079–0.086)	0.084 (0.081–0.087)	0.085 (0.081–0.088)	0.086 (0.083–0.090)	<.001
ȃBody roundness index	3.1 (2.6–3.5)	4.3 (4.0–4.5)	5.1 (4.9–5.3)	6.1 (5.8–6.4)	7.8 (7.2–8.9)	<.001
Current smoker, *n* (%)	292 (17.9)	254 (15.3)	240 (14.5)	196 (11.8)	208 (12.4)	<.001
Ischemic cause of HF, *n* (%)	906 (55.4)	1000 (60.3)	1036 (62.5)	1039 (62.8)	984 (58.8)	.02
**Duration of HF, *n* (%)**						<.001
ȃ<=1 year	624 (38.2)	528 (31.8)	470 (28.3)	430 (26.0)	436 (26.0)	
ȃ1–5 years	613 (37.5)	656 (39.5)	647 (39.0)	656 (39.6)	623 (37.2)	
ȃ> 5 years	398 (24.3)	475 (28.6)	541 (32.6)	569 (34.4)	615 (36.7)	
LVEF, mean (SD)	28.4 ± 6.2	29.2 ± 6.4	29.5 ± 6.2	30.0 ± 6.1	30.4 ± 6.1	<.001
**NYHA class at randomization, *n* (%)**						<.001
ȃI	85 (5.2)	104 (6.3)	70 (4.2)	63 (3.8)	55 (3.3)	
ȃII	1172 (71.8)	1188 (71.7)	1185 (71.5)	1210 (73.3)	1080 (64.6)	
ȃIII	366 (22.4)	356 (21.5)	396 (23.9)	364 (22.0)	517 (30.9)	
ȃIV	10 (0.6)	10 (0.6)	6 (0.4)	14 (0.8)	20 (1.2)	
KCCQ-OSS, mean (SD)	74.3 ± 18.6	75.2 ± 18.0	74.6 ± 18.9	73.4 ± 19.1	67.4 ± 21.1	<.001
KCCQ-CSS, mean (SD)	78.5 ± 18.3	78.5 ± 17.9	77.6 ± 18.4	75.9 ± 19.1	69.7 ± 21.0	<.001
**Medical history, *n* (%)**						
ȃHospitalization for HF	991 (60.6)	995 (60.0)	1054 (63.6)	1045 (63.1)	1121 (67.0)	<.001
ȃHypertension	935 (57.2)	1087 (65.5)	1164 (70.2)	1297 (78.4)	1382 (82.6)	<.001
ȃDiabetes	362 (22.1)	498 (30.0)	551 (33.2)	640 (38.7)	815 (48.7)	<.001
ȃHistory of atrial fibrillation	472 (28.9)	561 (33.8)	591 (35.6)	668 (40.4)	761 (45.5)	<.001
ȃAtrial fibrillation on electrocardiogram	304 (19.0)	385 (23.5)	370 (22.8)	440 (26.8)	513 (31.0)	<.001
ȃPrevious myocardial infarction	625 (38.2)	715 (43.1)	763 (46.0)	785 (47.4)	698 (41.7)	.004
ȃPrevious stroke	143 (8.7)	141 (8.5)	148 (8.9)	123 (7.4)	166 (9.9)	.55
ȃChronic obstructive pulmonary disease	193 (11.8)	180 (10.8)	209 (12.6)	221 (13.4)	264 (15.8)	<.001
ȃCancer	64 (3.9)	82 (4.9)	82 (4.9)	87 (5.3)	91 (5.4)	.046
**Treatment, *n* (%)**						
ȃBeta-blocker	1497 (91.6)	1526 (92.0)	1559 (94.0)	1539 (93.0)	1586 (94.7)	<.001
ȃMineralocorticoid-receptor antagonist	938 (57.4)	914 (55.1)	911 (54.9)	924 (55.8)	929 (55.5)	.44
ȃDiuretic	1247 (76.3)	1291 (77.8)	1304 (78.6)	1349 (81.5)	1458 (87.1)	<.001
ȃDigitalis	635 (38.8)	561 (33.8)	502 (30.3)	409 (24.7)	407 (24.3)	<.001
ȃAntiplatelet	906 (55.4)	933 (56.2)	995 (60.0)	933 (56.4)	908 (54.2)	.55
ȃOral anticoagulant	380 (23.2)	511 (30.8)	509 (30.7)	588 (35.5)	651 (38.9)	<.001
ȃStatin	740 (45.3)	903 (54.4)	1019 (61.5)	1031 (62.3)	966 (57.7)	<.001
ȃImplantable cardioverter-defibrillator	160 (9.8)	237 (14.3)	241 (14.5)	285 (17.2)	286 (17.1)	<.001
ȃCardiac resynchronization therapy	99 (6.1)	107 (6.4)	109 (6.6)	107 (6.5)	136 (8.1)	.03
Biomarkers, median (IQR) or mean (SD)^a^						
**NT-proBNP (pg/mL)**						
ȃNo atrial fibrillation on electrocardiogram	1934 (972–4360)	1644 (898–3546)	1481 (838–3003)	1306 (744–2425)	1213 (727–2212)	<.001
ȃAtrial fibrillation on electrocardiogram	2883 (1480–5583)	2109 (1331–4093)	2212 (1189–4145)	1803 (1107–3346)	1672 (1034–2859)	<.001
**BNP (pg/mL)**						
ȃNo atrial fibrillation on electrocardiogram	299 (160–637)	287 (167–548)	251 (155–465)	230 (143–392)	219 (136–390)	<.001
ȃAtrial fibrillation on electrocardiogram	329 (186–665)	277 (172–488)	262 (170–446)	230 (156–412)	215 (143–354)	<.001
**ucGMP/BNP ratio (nmol/pg)**						
ȃNo atrial fibrillation on electrocardiogram	3.4 (1.6–7.1)	4.2 (2.5–8.0)	4.7 (2.5–7.9)	4.6 (2.4–8.2)	4.6 (2.5–7.7)	.02
ȃAtrial fibrillation on electrocardiogram	3.8 (1.5–6.6)	6.7 (3.2–10.2)	5.6 (2.8–10.0)	5.9 (2.8–9.8)	6.3 (3.8–10.5)	.02
**Aldosterone (pmol/L)**						
ȃNo mineralocorticoid-receptor antagonist	208 (125–327)	199 (135–283)	190 (131–275)	228 (141–322)	231 (161–347)	.003
ȃMineralocorticoid-receptor antagonist	305 (171–522)	279 (156–458)	250 (178–483)	294 (164–469)	330 (208–558)	.04
Troponin T (ng/L)	16 (9–25)	13 (9–22)	15 (10–23)	14 (10–22)	15 (10–25)	.32
ST2 (ng/mL)	32 (24–43)	32 (27–42)	32 (25–41)	32 (26–42)	32 (25–41)	.72
MMP-9 (ng/mL)	56 (35–107)	62 (39–123)	63 (39–117)	59 (38–132)	75 (42–139)	.008
MMP-2 (ng/mL)	137 (115–162)	132 (113–151)	134 (117–152)	133 (115–157)	136 (118–159)	.22
TIMP1 (ng/mL)	130 (104–161)	122 (102–149)	122 (103–146)	122 (104–150)	128 (107–151)	.27
Galectin 3 (ng/mL)	17 (14–22)	17 (14–22)	17 (14–21)	17 (14–21)	18 (15–22)	.007
GDF-15 (ng/L)	1780 (1215–2434)	1620 (1130–2373)	1517 (1104–2246)	1622 (1200–2304)	1763 (1222–2573)	.14
Hemoglobin (g/L)	135 (125–147)	139 (129–150)	141 (130–151)	141 (130–152)	141 (130–152)	<.001
Hemoglobin A1c (%)	6.0 (5.7–6.5)	6.1 (5.7–6.7)	6.2 (5.8–6.8)	6.2 (5.8–6.9)	6.4 (5.9–7.4)	<.001
White blood cell count (10^9/L)	6.5 (5.3–7.8)	6.6 (5.4–7.8)	6.8 (5.7–8.1)	6.8 (5.7–8.1)	7.1 (6.0–8.4)	<.001
Neutrophil count (10^9/L)	3.9 (3.1–5.0)	4.1 (3.2–5.0)	4.2 (3.4–5.2)	4.3 (3.4–5.3)	4.5 (3.6–5.5)	<.001
Neutrophil/lymphocyte ratio	2.2 (1.6–3.1)	2.4 (1.7–3.3)	2.4 (1.8–3.1)	2.4 (1.8–3.2)	2.5 (1.8–3.4)	<.001
Monocyte count (10^9/L)	0.4 (0.3–0.6)	0.4 (0.3–0.6)	0.5 (0.4–0.6)	0.5 (0.4–0.6)	0.5 (0.4–0.6)	<.001
Lymphocyte count (10^9/L)	1.7 (1.4–2.2)	1.7 (1.3–2.2)	1.8 (1.4–2.2)	1.8 (1.4–2.3)	1.8 (1.4–2.3)	<.001
eGFR (mL/min/1.73m^2)	69 (56–83)	67 (54–79)	65 (53–78)	65 (53–79)	65 (52–78)	<.001
Creatinine (μmol/L)	92 (79–109)	95 (81–113)	96 (83–114)	95 (81–115)	95 (80–116)	<.001
Blood urea nitrogen (mmol/L)	6.4 (5.0–8.2)	6.8 (5.4–8.6)	6.8 (5.4–8.6)	6.8 (5.7–8.6)	7.1 (5.7–9.3)	<.001
Cystatin (mg/L)	1.2 (1.0–1.4)	1.1 (1.0–1.4)	1.1 (1.0–1.4)	1.2 (1.0–1.4)	1.2 (1.0–1.5)	.01
**Urine albumin-creatinine ratio (mg/mmol)**						
ȃNo diabetes	0.9 (0.0–2.8)	0.8 (0.2–2.1)	1.0 (0.3–2.8)	0.8 (0.3–2.9)	1.1 (0.3–2.9)	.19
ȃDiabetes	1.6 (0.3–9.6)	1.5 (0.7–4.9)	1.8 (0.8–7.1)	1.7 (0.6–7.3)	2.1 (0.7–7.8)	.08
KIM-1 (pg/mL)	126 (82–192)	115 (78–176)	123 (80–181)	125 (88–197)	151 (99–213)	<.001
Uric acid (μmol/L)	375 (315–458)	393 (327–476)	404 (333–482)	404 (339–488)	410 (333–500)	<.001
ALT (IU/L)	17 (13–23)	18 (14–24)	18 (14–24)	18 (14–24)	18 (14–24)	<.001
AST (IU/L)	22 (18–27)	21 (18–26)	21 (17–25)	20 (17–25)	20 (17–24)	<.001
Alkaline phosphatase (IU/L)	73 (59–91)	71 (58–89)	72 (57–90)	70 (57–86)	73 (59–90)	<.001
Total bilirubin (μmol/L)	9 (7–14)	10 (7–14)	9 (7–12)	9 (7–12)	9 (7–12)	<.001
Albumin (g/L)	43 (41–45)	43 (41–45)	43 (41–45)	43 (41–45)	43 (41–44)	<.001

BNP, B-type natriuretic peptide; CSS, clinical summary score; eGFR, estimated glomerular filtration rate; HF, heart failure; KCCQ, Kansas City Cardiomyopathy Questionnaire; LVEF, left ventricular ejection fraction; NYHA, New York Heart Association; NT-proBNP, *n*-terminal pro-B-type natriuretic peptide; OSS, overall summary score; SD, standard deviation; ucGMP, urinary cyclic guanosine monophosphate.

Randomization visit, except ST2, MMP2, MMP9, TIMP1, GDF-15, and KIM-1, which were collected at the screening visit. Number of patients with missing biomarkers are: NT-proBNP, *n* = 14; BNP, *n* = 59; ucGMP/BNP ratio, *n* = 6375; Aldosterone, *n* = 6381; troponin T, *n* = 6345; ST2, *n* = 6803; MMP-9, *n* = 6999; MMP-2, *n* = 6998; TIMP1, *n* = 6983; galectin 3, *n* = 6354; GDF-15, *n* = 6850; Hemoglobin, *n* = 274; hemoglobin A1c, *n* = 180; White blood cell count, *n* = 294; Neutrophil count, *n* = 421; neutrophil/lymphocyte ratio, *n* = 421; monocyte count, *n* = 421; lymphocyte count, *n* = 421; eGFR, *n* = 0; creatinine, *n* = 0; blood urea nitrogen, *n* = 161; cystatin, *n* = 6343; urine albumin-creatinine ratio, *n* = 6416; KIM-1, *n* = 6846; uric acid, *n* = 186; ALT, *n* = 219; AST, *n* = 236; alkaline phosphatase, *n* = 199; total bilirubin, *n* = 199; albumin, *n* = 179.

### Overlap between body mass index categories and waist-to-height ratio quintiles

The overlap between BMI categories and waist-to-height ratio quintiles is shown in *Table 2*. Of the 2397 patients not overweight or obese, according to BMI, only 1253 (52%) were in waist-to-height ratio quintile 1 (covering the range of waist-to-height ratio thought to be healthy). The remaining 1144 participants (48%) not overweight or obese, according to BMI, were in waist-to-height ratio quintiles 2–5 (mainly quintiles 2 and 3).

**Table 2:** Correlation between body mass index categories and waist-to-height ratio quintiles

	Underweight	Normal weight	Overweight	Obesity class I	Obesity II/III
	BMI <18.5	BMI 18.5–24.9	BMI 25–29.9	BMI 30–34.9	BMI ≥35
	*n* = 151	*n* = 2246	*n* = 3203	*n* = 1779	*n* = 901
WHtR quintile 1	129	1124	347	30	5
WHtR quintile 2	14	746	819	69	11
WHtR quintile 3	4	282	1055	295	21
WHtR quintile 4	2	71	745	715	122
WHtR quintile 5	2	23	237	670	742

Spearman correlation coefficient: 0.74.

There was less reclassification of patients classified as obese by BMI; of the 2680 patients categorized as in obesity class I or class II/III, 2249 (84%) were in the fourth or fifth quintile of waist-to-height ratio.

### Outcomes according to anthropometric measures

#### Body mass index

In the analyses of BMI by WHO category, the risk of the primary outcome did not differ significantly between patients with obesity and those with normal weight in the analysis adjusted for only region and treatment. However, the risk of death (whether due to cardiovascular or all causes), was significantly lower in overweight and obese patients; conversely, the risk of heart failure hospitalization was higher among individuals in obesity class II/III (*Table 3a*). After adjustment for prognostic variables, including NT-proBNP, the association between higher BMI and lower risk of death was eliminated and the association between higher BMI and higher risk of HF hospitalization was accentuated (*Table 3a*).

**Table 3a:** Outcomes according to body mass index

	Underweight	Normal weight	Overweight	Obesity class I	Obesity class II/III
	BMI <18.5	BMI 18.5–24.9	BMI 25–29.9	BMI 30–34.9	BMI ≥35
	*n* = 153	*n* = 2268	*n* = 3249	*n* = 1810	*n* = 909
**HF hospitalization or cardiovascular death**					
ȃ*n* (%)	38 (24.8)	556 (24.5)	764 (23.5)	432 (23.9)	239 (26.3)
ȃEvent rate per 100 person-years (95% CI)	12.5 (9.1–17.1)	12.1 (11.1–13.1)	11.2 (10.4–12.0)	11.3 (10.3–12.4)	12.6 (11.1–14.3)
ȃHR (95% CI)^a^	1.02 (0.73–1.42)	Reference	0.94 (0.84–1.06)	0.94 (0.83–1.08)	1.04 (0.88–1.21)
ȃHR (95% CI)^b^	1.20 (0.86–1.67)	Reference	0.90 (0.81–1.01)	0.86 (0.75–0.99)	0.92 (0.78–1.09)
ȃHR (95% CI)^c^	1.06 (0.76–1.47)	Reference	1.03 (0.92–1.16)	1.09 (0.95–1.25)	1.24 (1.05–1.48)
**HF hospitalization**					
ȃ*n* (%)	18 (11.8)	289 (12.7)	451 (13.9)	273 (15.1)	163 (17.9)
ȃEvent rate per 100 person-years (95% CI)	5.9 (3.7–9.4)	6.3 (5.6–7.0)	6.6 (6.0–7.3)	7.1 (6.3–8.0)	8.6 (7.4–10.0)
ȃHR (95% CI)^a^	0.96 (0.60–1.55)	Reference	1.04 (0.90–1.22)	1.10 (0.93–1.31)	1.26 (1.03–1.54)
ȃHR (95% CI)^b^	1.16 (0.72–1.88)	Reference	0.98 (0.84–1.14)	0.96 (0.81–1.15)	1.06 (0.86–1.32)
ȃHR (95% CI)^c^	1.03 (0.63–1.67)	Reference	1.12 (0.96–1.31)	1.22 (1.01–1.46)	1.43 (1.15–1.78)
**Cardiovascular death**					
ȃ*n* (%)	30 (19.6)	387 (17.1)	474 (14.6)	242 (13.4)	117 (12.9)
ȃEvent rate per 100 person-years (95% CI)	9.3 (6.5–13.3)	7.9 (7.1–8.7)	6.5 (5.9–7.1)	5.8 (5.1–6.6)	5.6 (4.6–6.7)
ȃHR (95% CI)^a^	1.14 (0.78–1.65)	Reference	0.85 (0.74–0.98)	0.77 (0.65–0.91)	0.75 (0.60–0.93)
ȃHR (95% CI)^b^	1.27 (0.87–1.86)	Reference	0.83 (0.73–0.96)	0.75 (0.63–0.89)	0.72 (0.58–0.91)
ȃHR (95% CI)^c^	1.11 (0.76–1.63)	Reference	0.96 (0.83–1.11)	0.96 (0.80–1.15)	1.00 (0.79–1.26)
**Non-cardiovascular death**					
ȃ*n* (%)	6 (3.9)	79 (3.5)	119 (3.7)	65 (3.6)	24 (2.6)
ȃEvent rate per 100 person-years (95% CI)	1.9 (0.8–4.1)	1.6 (1.3–2.0)	1.6 (1.4–1.9)	1.6 (1.2–2.0)	1.1 (0.8–1.7)
ȃHR (95% CI)^a^	1.47 (0.64–3.39)	Reference	0.88 (0.66–1.17)	0.81 (0.58–1.13)	0.57 (0.36–0.91)
ȃHR (95% CI)^b^	1.75 (0.75–4.08)	Reference	0.87 (0.65–1.16)	0.83 (0.59–1.18)	0.64 (0.39–1.06)
ȃHR (95% CI)^c^	1.70 (0.73–3.98)	Reference	0.91 (0.68–1.23)	0.91 (0.64–1.30)	0.72 (0.44–1.19)
**All-cause death**					
ȃ*n* (%)	36 (23.5)	466 (20.5)	593 (18.3)	307 (17.0)	141 (15.5)
ȃEvent rate per 100 person-years (95% CI)	11.2 (8.1–15.5)	9.5 (8.7–10.4)	8.1 (7.5–8.8)	7.4 (6.6–8.3)	6.7 (5.7–7.9)
ȃHR (95% CI)^a^	1.18 (0.84–1.67)	Reference	0.86 (0.75–0.97)	0.78 (0.67–0.90)	0.71 (0.58–0.86)
ȃHR (95% CI)^b^	1.33 (0.94–1.88)	Reference	0.84 (0.74–0.95)	0.76 (0.65–0.89)	0.71 (0.57–0.87)
ȃHR (95% CI)^c^	1.19 (0.84–1.69)	Reference	0.95 (0.84–1.08)	0.95 (0.81–1.11)	0.94 (0.76–1.16)

BMI, body mass index; CI, confidence interval; HF, heart failure; HR, hazard ratio.

Adjusted for treatment and region.

Adjusted for treatment, age, sex, region, systolic blood pressure, heart rate, estimated glomerular filtration rate, left ventricular ejection fraction, NYHA functional class, HF etiology, duration of HF, prior HF hospitalization, a history of diabetes, and atrial fibrillation.

Adjusted for the above-mentioned variables and log of NT-proBNP.

**Figure 3:**
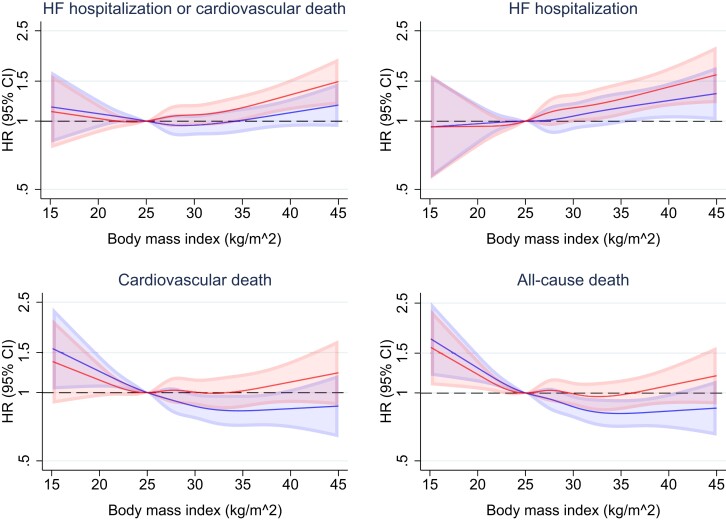
Outcomes according to body mass index. This figure shows the risk of heart failure hospitalization or cardiovascular death, its components, and all-cause death, according to continuous body mass index. The solid line represents the hazard ratio and the shaded area the 95% CI. The reference is a body mass index of 25 kg/m^2^. The blue spline is adjusted for treatment and region. The red spline is adjusted for treatment, age, sex, region, systolic blood pressure, heart rate, estimated glomerular filtration rate, left ventricular ejection fraction, log of *n*-terminal pro-B-type natriuretic peptide, New York Heart Association functional class, heart failure aetiology, duration of heart failure, prior heart failure hospitalization, a history of diabetes, and atrial fibrillation. CI, confidence interval; HF, heart failure; HR, hazard ratio.

When BMI was examined as a continuous variable, a similar picture was evident although the small ‘underweight’ BMI category appeared to be associated with a higher risk of death from non-cardiovascular causes (*Figure 3*).

#### Waist-to-height ratio

In the analyses of waist-to-height ratio by quintile, the risk of the primary outcome did not differ significantly between patients in quintile 5, compared to those in quintile 1, in the analysis adjusted for only region and treatment (*Table 3b*). However, the risk of death (whether due to cardiovascular or all causes), tended to be lower in patients in quintile 5, although this was not as clear as for BMI. Conversely, the risk of HF hospitalization was significantly higher in individuals in quintile 5, compared with quintile 1. After adjustment for prognostic variables, the association between higher waist-to-height ratio and lower risk of death was eliminated and the association between higher waist-to-height ratio and higher risk of HF hospitalization persisted (*Table 3*).

**Table 3b:** Outcomes according to quintile of waist-to-height ratio

	Quintile 1	Quintile 2	Quintile 3	Quintile 4	Quintile 5
	*n* = 1635	*n* = 1659	*n* = 1658	*n* = 1655	*n* = 1674
**HF hospitalization or cardiovascular death**					
ȃ*n* (%)	378 (23.1)	378 (22.8)	433 (26.1)	390 (23.6)	425 (25.4)
ȃEvent rate per 100 person-years (95% CI)	11.4 (10.3–12.6)	10.9 (9.8–12.0)	12.4 (11.3–13.6)	11.2 (10.1–12.4)	12.3 (11.1–13.5)
ȃHR (95% CI)^a^	Reference	0.98 (0.85–1.13)	1.13 (0.98–1.30)	1.02 (0.88–1.18)	1.12 (0.97–1.29)
ȃHR (95% CI)^b^	Reference	1.00 (0.86–1.16)	1.18 (1.01–1.39)	1.12 (0.93–1.33)	1.18 (0.96–1.44)
ȃHR (95% CI)^c^	Reference	1.02 (0.88–1.19)	1.24 (1.06–1.46)	1.18 (0.99–1.41)	1.27 (1.03–1.55)
**HF hospitalization**					
ȃ*n* (%)	198 (12.1)	208 (12.5)	257 (15.5)	230 (13.9)	284 (17.0)
ȃEvent rate per 100 person-years (95% CI)	6.0 (5.2–6.9)	6.0 (5.2–6.9)	7.4 (6.5–8.3)	6.6 (5.8–7.5)	8.2 (7.3–9.2)
ȃHR (95% CI)^a^	Reference	1.02 (0.84–1.24)	1.26 (1.04–1.52)	1.12 (0.92–1.37)	1.37 (1.13–1.65)
ȃHR (95% CI)^b^	Reference	1.01 (0.83–1.24)	1.24 (1.00–1.54)	1.13 (0.89–1.43)	1.30 (1.00–1.69)
ȃHR (95% CI)^c^	Reference	1.04 (0.85–1.28)	1.31 (1.05–1.62)	1.19 (0.93–1.51)	1.39 (1.06–1.81)
**Cardiovascular death**					
ȃ*n* (%)	263 (16.1)	248 (14.9)	263 (15.9)	240 (14.5)	224 (13.4)
ȃEvent rate per 100 person-years (95% CI)	7.5 (6.6–8.4)	6.7 (5.9–7.6)	7.0 (6.2–7.9)	6.4 (5.6–7.3)	5.9 (5.2–6.7)
ȃHR (95% CI)^a^	Reference	0.93 (0.78–1.10)	0.98 (0.82–1.17)	0.91 (0.76–1.09)	0.85 (0.70–1.02)
ȃHR (95% CI)^b^	Reference	0.98 (0.82–1.18)	1.09 (0.90–1.33)	1.11 (0.89–1.39)	1.06 (0.82–1.38)
ȃHR (95% CI)^c^	Reference	1.01 (0.84–1.21)	1.16 (0.95–1.41)	1.19 (0.95–1.49)	1.15 (0.88–1.49)
**Non-cardiovascular death**					
ȃ*n* (%)	58 (3.5)	55 (3.3)	58 (3.5)	60 (3.6)	55 (3.3)
ȃEvent rate per 100 person-years (95% CI)	1.6 (1.3–2.1)	1.5 (1.1–1.9)	1.5 (1.2–2.0)	1.6 (1.2–2.1)	1.4 (1.1–1.9)
ȃHR (95% CI)^a^	Reference	0.82 (0.56–1.19)	0.80 (0.55–1.16)	0.82 (0.56–1.18)	0.71 (0.49–1.04)
ȃHR (95% CI)^b^	Reference	0.82 (0.56–1.21)	0.81 (0.53–1.23)	0.86 (0.55–1.36)	0.87 (0.52–1.48)
ȃHR (95% CI)^c^	Reference	0.83 (0.57–1.22)	0.82 (0.54–1.25)	0.88 (0.56–1.39)	0.90 (0.53–1.52)
**All-cause death**					
ȃ*n* (%)	321 (19.6)	303 (18.3)	321 (19.4)	300 (18.1)	279 (16.7)
ȃEvent rate per 100 person-years (95% CI)	9.1 (8.2–10.2)	8.2 (7.3–9.2)	8.5 (7.6–9.5)	8.0 (7.2–9.0)	7.3 (6.5–8.2)
ȃHR (95% CI)^a^	Reference	0.91 (0.77–1.06)	0.94 (0.81–1.11)	0.89 (0.76–1.05)	0.82 (0.69–0.97)
ȃHR (95% CI)^b^	Reference	0.95 (0.81–1.12)	1.04 (0.87–1.24)	1.06 (0.87–1.30)	1.03 (0.81–1.30)
ȃHR (95% CI)^c^	Reference	0.97 (0.83–1.15)	1.09 (0.91–1.31)	1.13 (0.92–1.38)	1.10 (0.87–1.39)

CI, confidence interval; HF, heart failure; HR, hazard ratio.

Adjusted for treatment and region.

Adjusted for treatment, age, sex, region, systolic blood pressure, heart rate, estimated glomerular filtration rate, left ventricular ejection fraction, body mass index, NYHA functional class, HF etiology, duration of HF, prior HF hospitalization, a history of diabetes, and atrial fibrillation.

Adjusted for the above-mentioned variables and log of NT-proBNP.

**Figure 4:**
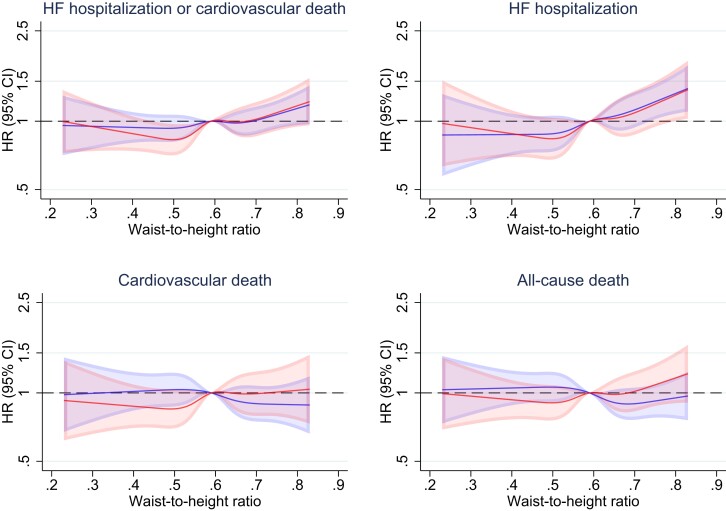
Outcomes according to waist-to-height ratio. This figure shows the risk of heart failure hospitalization or cardiovascular death, its components, and all-cause death, according to continuous waist-to-height ratio. The solid line represents the hazard ratio and the shaded area the 95% CI. The reference is the median waist-to-height ratio (0.58). The blue spline is adjusted for treatment and region. The red spline is adjusted for treatment, age, sex, region, systolic blood pressure, heart rate, estimated glomerular filtration rate, left ventricular ejection fraction, log of *n*-terminal pro-B-type natriuretic peptide, body mass index, New York Heart Association functional class, heart failure aetiology, duration of heart failure, prior heart failure hospitalization, a history of diabetes, and atrial fibrillation. CI, confidence interval; HF, heart failure; HR, hazard ratio.

When waist-to-height ratio was examined as a continuous variable, a similar picture was evident although a low waist-to-height ratio did not appear to be associated with a higher risk of death from non-cardiovascular causes (*Figure 4*).

#### Other anthropometric measures

The association between the other anthropometric measures examined as continuous variables and outcomes are shown in Supplementary material online, *Figures S1–S7*. The association between waist-to-hip ratio examined as a continuous variable and outcomes in men and women, respectively, are shown in Supplementary material online, *Figures S8* and *S9*. After adjustment for prognostic variables, greater adiposity, as assessed by body roundness index and relative fat mass, was associated with a higher risk of HF hospitalization. None of the anthropometric measures (i.e. waist circumference, waist-to-hip ratio, body shape index, weight-adjusted-waist index, and body surface area) was associated with cardiovascular death.

### Effects of sacubitril/valsartan according to anthropometric measures

#### Body mass index

Compared with enalapril, sacubitril/valsartan reduced the risk of HF hospitalization or cardiovascular death across BMI categories (*P* for interaction = 0.50) (*Table 4*). The beneficial effect of sacubitril/valsartan was consistent across BMI categories for HF hospitalization (*P* for interaction = .41), cardiovascular death (*P* for interaction = .81), and death from any cause (*P* for interaction = .97) (*Table 4*).

**Table 4:** Effects of sacubitril/valsartan compared with enalapril according to body mass index

	Underweight	Normal weight	Overweight	Obesity class I	Obesity class II/III	*P*-value for interaction
	BMI <18.5	BMI 18.5–24.9	BMI 25–29.9	BMI 30–34.9	BMI ≥35	
	*n* = 153	*n* = 2268	*n* = 3249	*n* = 1810	*n* = 909	
	HR (95% CI)	HR (95% CI)	HR (95% CI)	HR (95% CI)	HR (95% CI)	
HF hospitalization or cardiovascular death	0.80 (0.42–1.53)	0.91 (0.77–1.07)	0.79 (0.68–0.91)	0.75 (0.62–0.90)	0.70 (0.54–0.91)	.50
HF hospitalization	0.93 (0.37–2.38)	0.97 (0.77–1.22)	0.75 (0.62–0.90)	0.74 (0.59–0.95)	0.71 (0.52–0.97)	.41
Cardiovascular death	0.79 (0.38–1.66)	0.88 (0.72–1.07)	0.80 (0.67–0.96)	0.72 (0.55–0.92)	0.73 (0.50–1.06)	.81
All-cause death	0.81 (0.41–1.60)	0.90 (0.75–1.08)	0.82 (0.70–0.96)	0.83 (0.66–1.04)	0.81 (0.58–1.13)	.97

BMI, body mass index; CI, confidence interval; HF, heart failure; HR hazard ratio.

Models adjusted for region.

#### Waist-to-height ratio

The beneficial effect of sacubitril/valsartan was also consistent for the primary and secondary endpoints according to quintiles of waist-to-height ratio (see Supplementary material online, *Table S1*).

## Discussion

Among the patients with HFrEF in PARADIGM-HF, there was no longer evidence of a BMI-related ‘obesity-survival paradox’ after comprehensive adjustment for other prognostic variables. Moreover, the counterintuitive epidemiologic observation of a lower risk of death in patients with greater adiposity was less apparent with the newer anthropometric indices. All anthropometric indices examined showed that greater adiposity was associated with a higher risk of HF hospitalization as this was more evident with the newer indices (*Structured Graphical Abstract*).

In the minimally adjusted analyses (adjusted for only region and treatment assignment), overweight and obesity, defined using conventional BMI categories, were associated with a lower risk of death from any cause, and cardiovascular causes, compared with normal weight, as has been reported previously.^4–8,37–40^ However, most prior studies did not adjust for other prognostic variables which vary greatly across BMI categories, particularly the most powerful of these, i.e. natriuretic peptides.^4–8,26–34,37–40^ We were able to adjust for a broad range of prognostic variables, including NT-proBNP, and thereby minimize the impact of any potential residual confounding. After this adjustment, the ‘survival paradox’ related to high BMI was eliminated. Furthermore, none of the newer anthropometric measures showed the same association between greater adiposity and a lower risk of cardiovascular and all-cause death as was seen with BMI when adjusted for conventional risk variables (but not NT-proBNP); additional adjustment for NT-proBNP eliminated any suggestion of an ‘obesity-survival paradox’ with BMI. Indeed, when each of these indices was analyzed as a continuous variable their relationship with death was entirely flat. It is not certain why the newer indices showed a weaker relationship between adiposity and fatal outcomes, compared with BMI. However, an inspection of patient characteristics and biomarkers suggested a somewhat steeper gradient in age, atrial fibrillation, and NYHA class, as well as natriuretic peptide, aldosterone, and hemoglobin levels across BMI categories (normal to obesity class II/III) compared with the other indices (lowest to highest quintile). This may also explain why adjustment for other prognostic variables changed the association between higher adiposity and risk of death more for BMI than the other anthropometric indices.

Importantly, three of the newer anthropometric indices (waist-to-height ratio, relative fat mass, and body roundness index) demonstrated a significantly higher risk of HF hospitalization (and the composite of HF hospitalization or death from cardiovascular causes) in patients with greater adiposity, an association that was less apparent with BMI. These newer indices incorporate waist circumference and height, with the former better reflecting intra-abdominal fat (‘central obesity’) and the latter accounting for sex- and race-based differences in stature and skeletal weight. Notably, neither newer index included measured overall weight, the interpretation of which in patients with HF may be confounded by fluid retention or unintentional weight loss due to other illness. Interestingly, despite the steeper gradient in prognostic variables across BMI categories, compared to waist-to-height ratio quintiles, described above, the association between higher BMI and HF hospitalization was not as strong as that between higher waist-to-height ratio and HF hospitalization. This suggests that these new anthropometric indices identify pathophysiologic processes not reflected by conventional prognostic variables e.g. related to the distribution of body fat.

Different associations with outcomes were seen at the lower end of the range of adiposity and these also varied between the anthropometric indices. When considered as a continuous variable, a very low BMI was associated with a higher risk of death, which has been shown previously. However, we demonstrated that this was explained by a significant excess of non-cardiovascular death rather than cardiovascular death. The excess risk persisted after adjustment for other prognostic variables. This relationship was not as clearly evident with waist-to-height ratio (or the other newer anthropometric indices).

The relationship between adiposity and health-related quality of life (measured with the Kansas City Cardiomyopathy Questionnaire) was consistent between BMI and waist-to-height ratio. Both showed a steep decline in health-related quality of life with increasing adiposity, a relationship which was consistent with the relationship between waist-to-height ratio and HF hospitalization but opposite to that for BMI and mortality (in an unadjusted analysis).

Collectively these data show that greater adiposity in HFrEF is associated with a higher symptom burden, worse quality of life, and a greater risk of HF hospitalization. Other studies have shown that obese patients also have a higher risk of developing diabetes, atrial fibrillation, sleep apnea, and other comorbid conditions, compared with non-obese patients.^26,34,41–44^ Also, BMI > 35 kg/m^2^ is a contraindication to heart transplantation.^45^ Therefore, there is a strong rationale for promoting weight loss in obese patients especially as the ‘obesity-survival paradox’ seems to be an artifact of unadjusted analyses of BMI. Unfortunately, few randomized controlled trials using dietary and exercise intervention, bariatric surgery, or novel pharmacological therapies have been conducted in patients with HFrEF, although the latter are being investigated in individuals with HFpEF.^46–52^ The 2021 European Society of Cardiology and 2022 American College of Cardiology/American Heart Association guidelines do not provide any recommendation regarding weight management in HFrEF.^45,53^ Efforts to find effective and safe approaches to reducing weight in patients with HFrEF are therefore warranted.

### Effect of sacubitril/valsartan according to anthropometric measures

The effect of HF therapies according to anthropometric measures is also of interest because of the hypothesis that the ‘obesity-survival paradox’ could, to some extent, reflect a greater effect of treatment in obese patients. Support for this was provided by a *post hoc* analysis of the Eplerenone in Mild Patients Hospitalization and Survival Study in Heart Failure (EMPHASIS-HF) trial, in which the benefit of the mineralocorticoid receptor antagonist, eplerenone, appeared to be greater in patients with larger waist circumference.^54^ However, such a relationship has not been established for other effective therapies in HFrEF, and a recent analysis of the dapagliflozin and prevention of adverse outcomes in heart failure (DAPA-HF) trial demonstrated that the benefit of dapagliflozin was consistent across the spectrum of BMI.^34^ In the present analysis, the beneficial effect of sacubitril/valsartan, compared with enalapril, was consistent for all outcomes across the spectrum of BMI and waist-to-height ratio. Specifically, there was no evidence of a diminished benefit in underweight patients or a larger benefit in obese patients.

### Study limitations

This study has some limitations. Due to the observational nature of this study, the possibility of unmeasured confounding, despite adjustment for known prognostic variables, remains. Abdominal anthropometric measurements, such as waist circumference, are associated with higher measurement error than BMI, especially when these measurements are performed by different individuals.^55^ In addition, the analyses on the association between anthropometric measures at randomization and clinical adverse outcomes did not account for any change in e.g. weight or waist circumference during follow-up. Previous studies have suggested that the ‘obesity-survival paradox’ in HF may be affected by the level of cardiorespiratory fitness.^56–59^ Although it would have been interesting to examine the association between anthropometric measures and outcomes according to cardiorespiratory fitness levels, these data were not available. Finally, only 153 patients had a BMI <18.5 kg/m^2^ (the ‘underweight’ BMI category) and 171 patients a waist-to-height ratio <0.40, and our findings clearly cannot be extrapolated to patients with a low BMI or waist-to-height ratio.

## Conclusion

In a large cohort of patients with HFrEF, the ‘obesity-survival paradox’ related to BMI was eliminated by comprehensive adjustment for prognostic variables. Importantly, alternative anthropometric measurements showed less evidence of this paradox, and two indices that incorporate waist circumference and height, but not weight, showed a clearer association between greater adiposity and a higher risk of HF hospitalization. Greater adiposity was associated with worse symptoms and health-related quality of life, irrespective of the anthropometric index used. These findings collectively suggest a need to test the potential benefits of intentional weight loss in patients living with obesity and HFrEF.

## Supplementary Material

ehad083_Supplementary_Data

## Data Availability

Trial data will be made available by the sponsor, Novartis, in accordance with their data sharing policy.
