# Supplementary material for: Anthropometric measures and adverse outcomes in heart failure with reduced ejection fraction: revisiting the obesity paradox
Source: Eur Heart J. 2023 Mar 22;44(13):1136–53. doi: 10.1093/eurheartj/ehad083 (PMC10111968; doi:10.1093/eurheartj/ehad083)
Supplement: ehad083_Supplementary_Data [file ehad083_supplementary_data.docx]

**Supplementary Table 1. Effects of sacubitril/valsartan compared with enalapril according to quintile of waist-to-height ratio**

|  | Quintile 1  N=1,635  HR (95% CI) | Quintile 2  N=1,659  HR (95% CI) | Quintile 3  N=1,658  HR (95% CI) | Quintile 4  N=1,655  HR (95% CI) | Quintile 5  N=1,674  HR (95% CI) | P-value for interaction |
| --- | --- | --- | --- | --- | --- | --- |
| HF hospitalization or cardiovascular death | 0.79 (0.65-0.97) | 0.87 (0.71-1.07) | 0.79 (0.65-0.95) | 0.72 (0.59-0.87) | 0.77 (0.64-0.94) | 0.72 |
| HF hospitalization | 0.83 (0.63-1.09) | 0.79 (0.6-1.03) | 0.75 (0.59-0.96) | 0.76 (0.59-0.99) | 0.79 (0.62-1) | 0.99 |
| Cardiovascular death | 0.75 (0.59-0.96) | 0.98 (0.76-1.25) | 0.82 (0.64-1.04) | 0.72 (0.56-0.93) | 0.73 (0.56-0.95) | 0.41 |
| All-cause death | 0.79 (0.64-0.99) | 0.94 (0.75-1.17) | 0.91 (0.73-1.14) | 0.72 (0.58-0.91) | 0.82 (0.65-1.04) | 0.50 |

*CI, confidence interval; HF, heart failure; HR hazard ratio.*

*Models adjusted for region.*

**Supplementary Figure 1.** **Outcomes according to waist circumference**


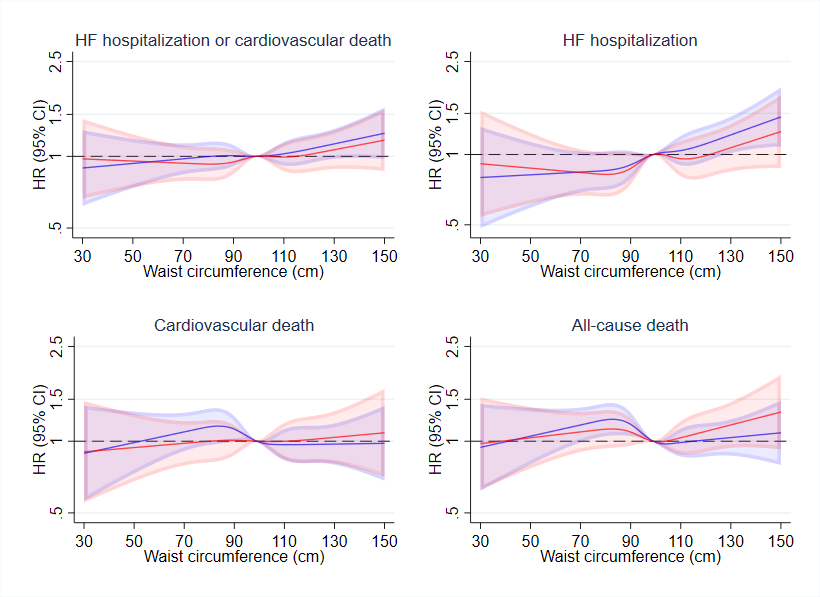


*This figure shows the risk of heart failure hospitalization or cardiovascular death, its components, and all-cause death, according to continuous waist circumference. The solid line represents the hazard ratio and the shaded area the 95% CI. The reference is the median waist circumference.*

*The blue spline is adjusted for treatment and region. The red spline is adjusted for treatment, age, sex, region, systolic blood pressure, heart rate, estimated glomerular filtration rate, left ventricular ejection fraction, log of N-terminal pro-B-type natriuretic peptide, body mass index, New York Heart Association functional class, heart failure etiology, duration of heart failure, prior heart failure hospitalization, a history of diabetes, and atrial fibrillation.*

*CI, confidence interval; HF, heart failure; HR, hazard ratio.***Supplementary Figure 2. Outcomes according to waist-to-hip ratio**


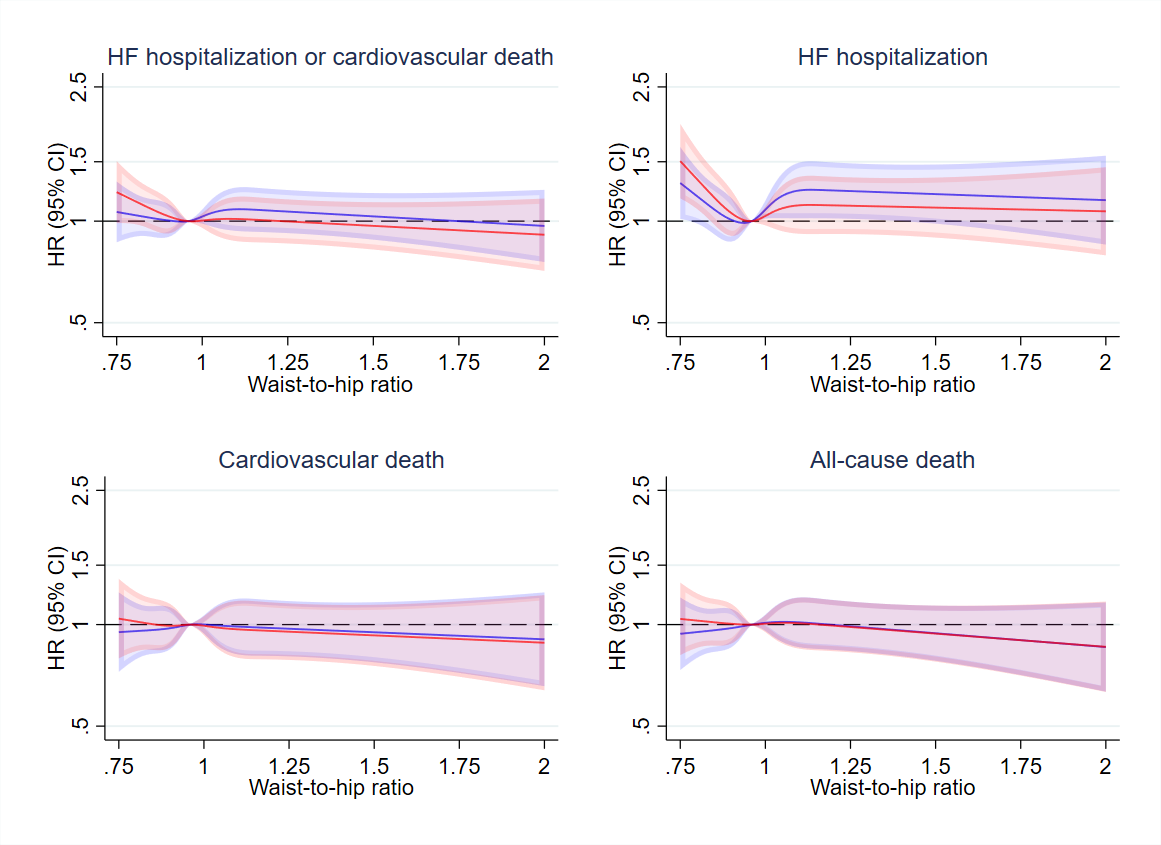


*This figure shows the risk of heart failure hospitalization or cardiovascular death, its components, and all-cause death, according to continuous waist-to-hip ratio. The solid line represents the hazard ratio and the shaded area the 95% CI. The reference is the median waist-to-hip ratio.*

*The blue spline is adjusted for treatment and region. The red spline is adjusted for treatment, age, sex, region, systolic blood pressure, heart rate, estimated glomerular filtration rate, left ventricular ejection fraction, log of N-terminal pro-B-type natriuretic peptide, body mass index, New York Heart Association functional class, heart failure etiology, duration of heart failure, prior heart failure hospitalization, a history of diabetes, and atrial fibrillation.*

*CI, confidence interval; HF, heart failure; HR, hazard ratio.*

**Supplementary Figure 3. Outcomes according to relative fat mass**


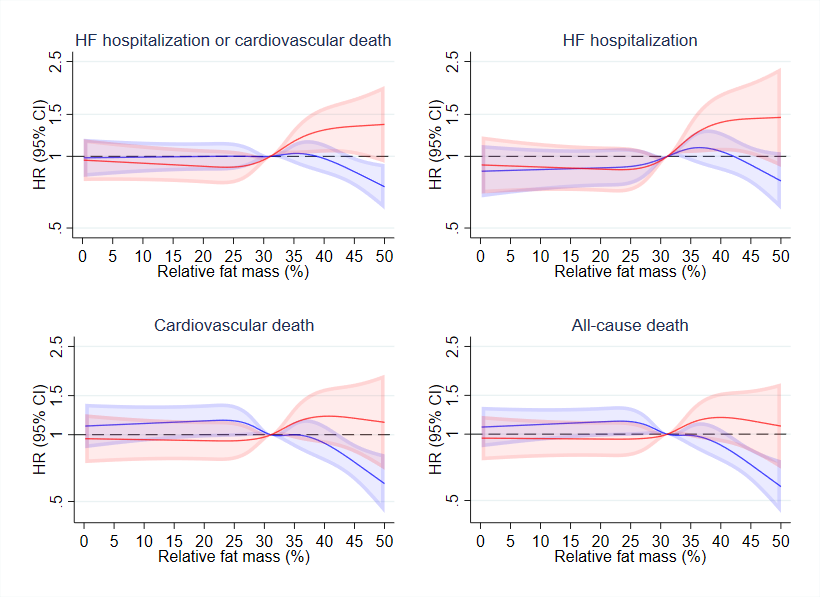


*This figure shows the risk of heart failure hospitalization or cardiovascular death, its components, and all-cause death, according to continuous relative fat mass. The solid line represents the hazard ratio and the shaded area the 95% CI. The reference is the median relative fat mass.*

*The blue spline is adjusted for treatment and region. The red spline is adjusted for treatment, age, sex, region, systolic blood pressure, heart rate, estimated glomerular filtration rate, left ventricular ejection fraction, log of N-terminal pro-B-type natriuretic peptide, body mass index, New York Heart Association functional class, heart failure etiology, duration of heart failure, prior heart failure hospitalization, a history of diabetes, and atrial fibrillation.*

*CI, confidence interval; HF, heart failure; HR, hazard ratio.*

**Supplementary Figure 4. Outcomes according to body roundness index**


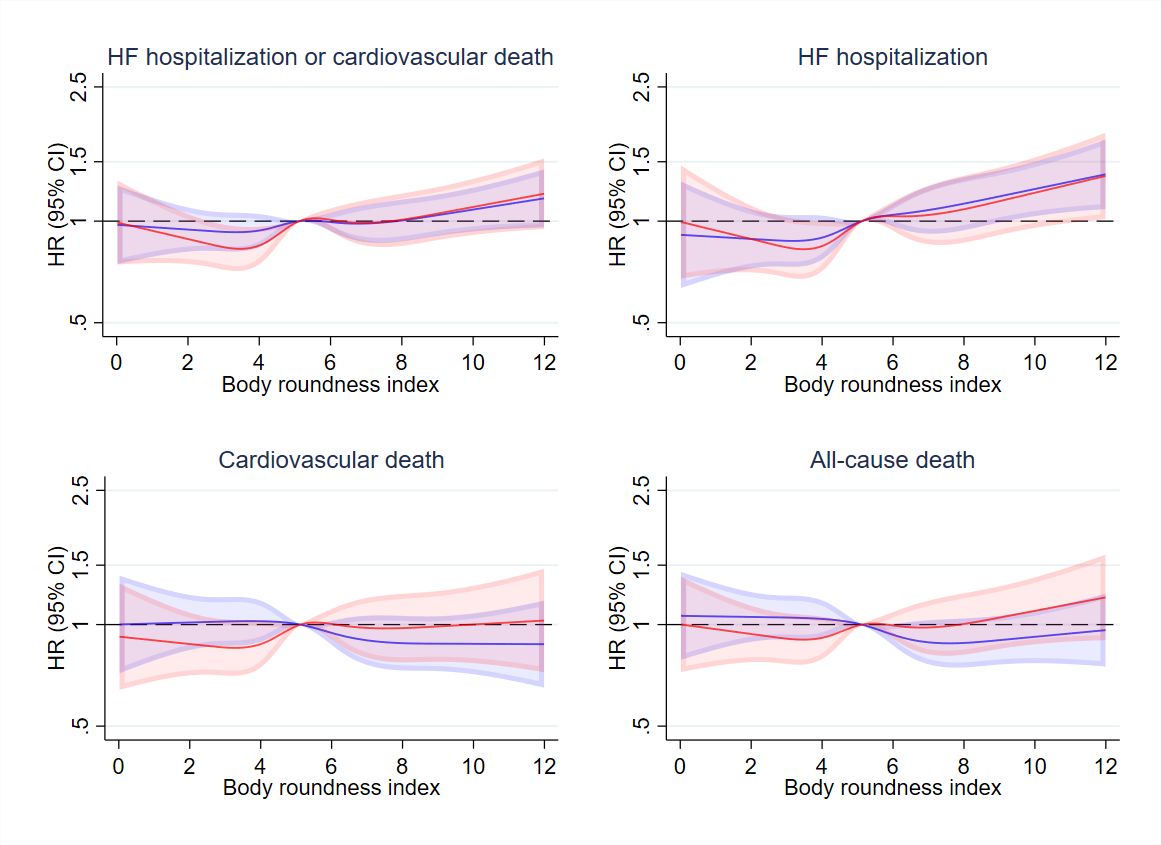


*This figure shows the risk of heart failure hospitalization or cardiovascular death, its components, and all-cause death, according to continuous body roundness index. The solid line represents the hazard ratio and the shaded area the 95% CI. The reference is the median body roundness index.*

*The blue spline is adjusted for treatment and region. The red spline is adjusted for treatment, age, sex, region, systolic blood pressure, heart rate, estimated glomerular filtration rate, left ventricular ejection fraction, log of N-terminal pro-B-type natriuretic peptide, body mass index, New York Heart Association functional class, heart failure etiology, duration of heart failure, prior heart failure hospitalization, a history of diabetes, and atrial fibrillation.*

*CI, confidence interval; HF, heart failure; HR, hazard ratio.*

**Supplementary Figure 5. Outcomes according to body shape index**


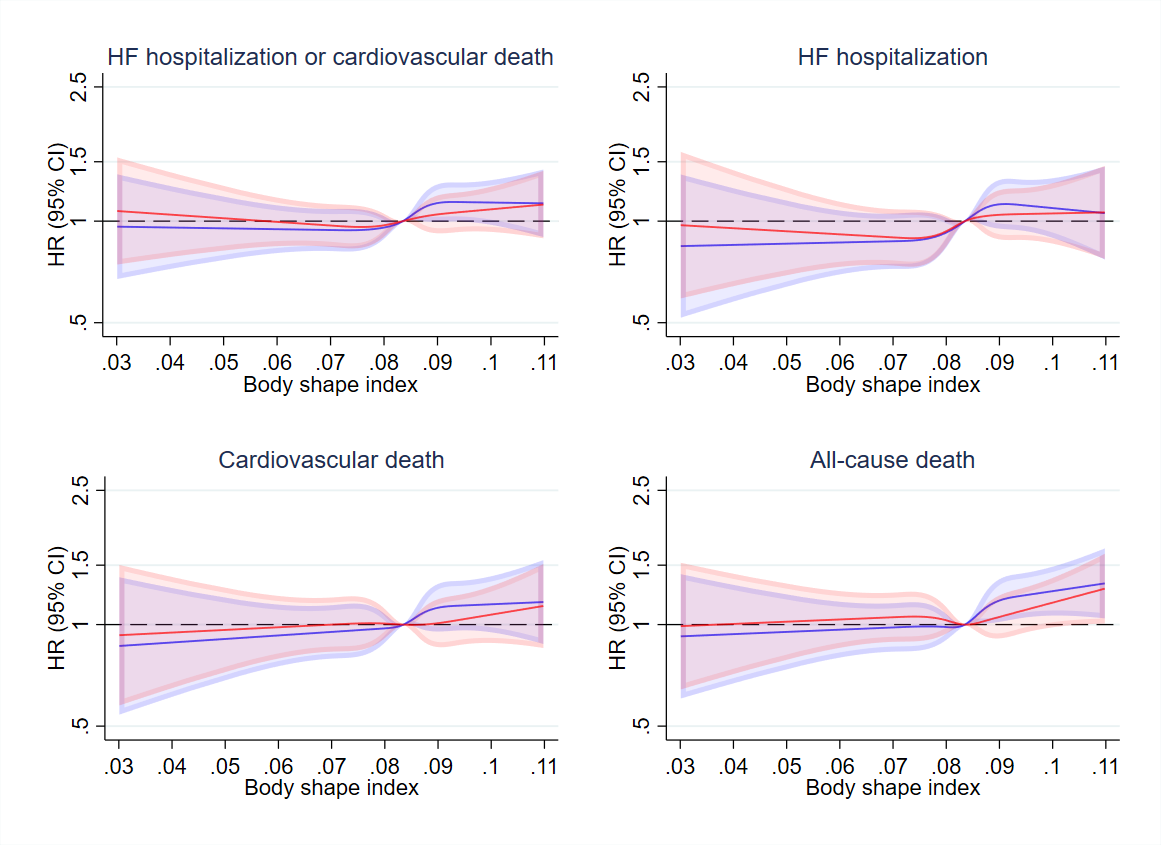


*This figure shows the risk of heart failure hospitalization or cardiovascular death, its components, and all-cause death, according to continuous body shape index. The solid line represents the hazard ratio and the shaded area the 95% CI. The reference is the median body shape index.*

*The blue spline is adjusted for treatment and region. The red spline is adjusted for treatment, age, sex, region, systolic blood pressure, heart rate, estimated glomerular filtration rate, left ventricular ejection fraction, log of N-terminal pro-B-type natriuretic peptide, body mass index, New York Heart Association functional class, heart failure etiology, duration of heart failure, prior heart failure hospitalization, a history of diabetes, and atrial fibrillation.*

*CI, confidence interval; HF, heart failure; HR, hazard ratio.*

**Supplementary Figure 6. Outcomes according to weight-adjusted-waist index**


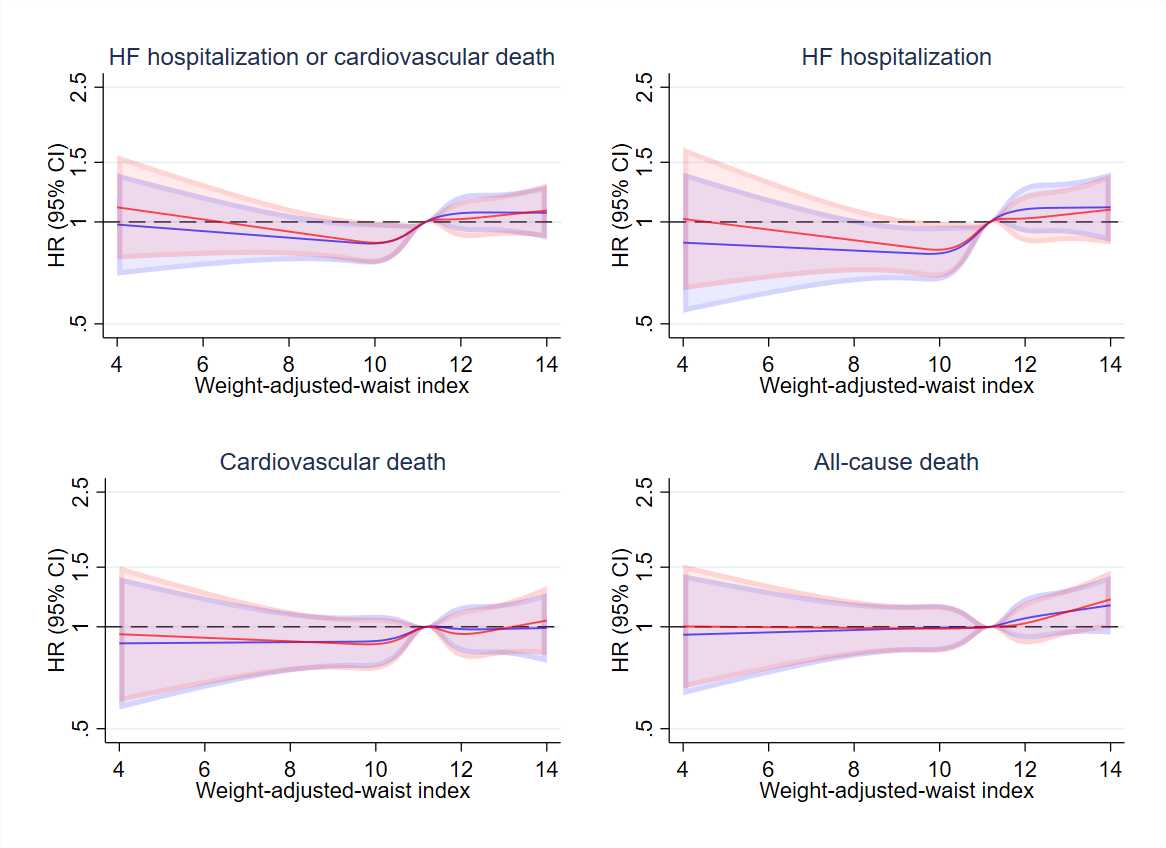


*This figure shows the risk of heart failure hospitalization or cardiovascular death, its components, and all-cause death, according to continuous weight-adjusted-waist index. The solid line represents the hazard ratio and the shaded area the 95% CI. The reference is the median weight-adjusted-waist index.*

*The blue spline is adjusted for treatment and region. The red spline is adjusted for treatment, age, sex, region, systolic blood pressure, heart rate, estimated glomerular filtration rate, left ventricular ejection fraction, log of N-terminal pro-B-type natriuretic peptide, body mass index, New York Heart Association functional class, heart failure etiology, duration of heart failure, prior heart failure hospitalization, a history of diabetes, and atrial fibrillation.*

*CI, confidence interval; HF, heart failure; HR, hazard ratio.*

**Supplementary Figure 7. Outcomes according to body surface area**


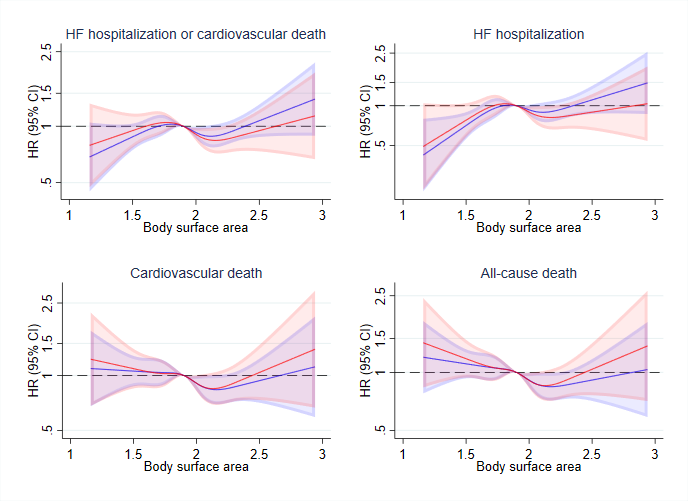


*This figure shows the risk of heart failure hospitalization or cardiovascular death, its components, and all-cause death, according to continuous body surface area. The solid line represents the hazard ratio and the shaded area the 95% CI. The reference is the median body surface area.*

*The blue spline is adjusted for treatment and region. The red spline is adjusted for treatment, age, sex, region, systolic blood pressure, heart rate, estimated glomerular filtration rate, left ventricular ejection fraction, log of N-terminal pro-B-type natriuretic peptide, body mass index, New York Heart Association functional class, heart failure etiology, duration of heart failure, prior heart failure hospitalization, a history of diabetes, and atrial fibrillation.*

*CI, confidence interval; HF, heart failure; HR, hazard ratio.*

**Supplementary Figure 8. Outcomes according to waist-to-hip ratio in men**


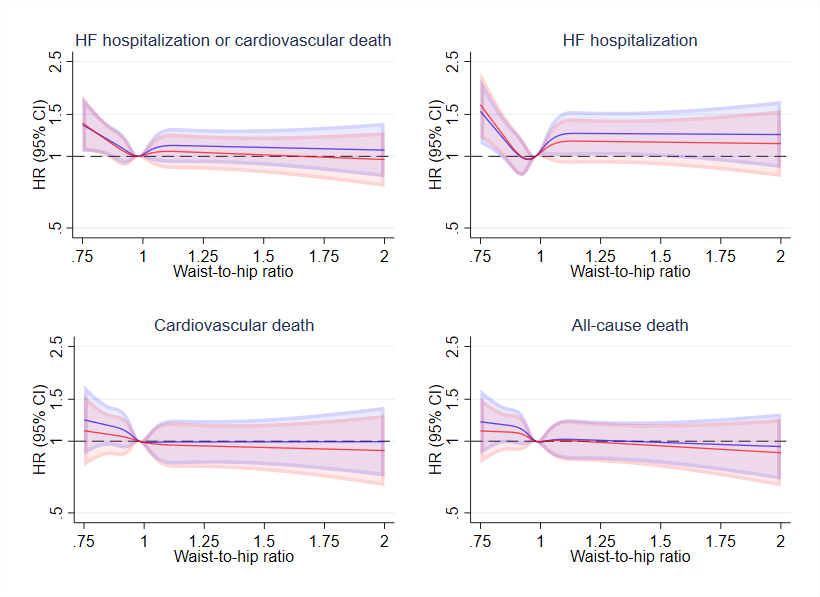


*This figure shows the risk of heart failure hospitalization or cardiovascular death, its components, and all-cause death, according to continuous waist-to-hip ratio. The solid line represents the hazard ratio and the shaded area the 95% CI. The reference is the median waist-to-hip ratio.*

*The blue spline is adjusted for treatment and region. The red spline is adjusted for treatment, age, region, systolic blood pressure, heart rate, estimated glomerular filtration rate, left ventricular ejection fraction, log of N-terminal pro-B-type natriuretic peptide, body mass index, New York Heart Association functional class, heart failure etiology, duration of heart failure, prior heart failure hospitalization, a history of diabetes, and atrial fibrillation.*

*CI, confidence interval; HF, heart failure; HR, hazard ratio.*

**Supplementary Figure 9. Outcomes according to waist-to-hip ratio in women**


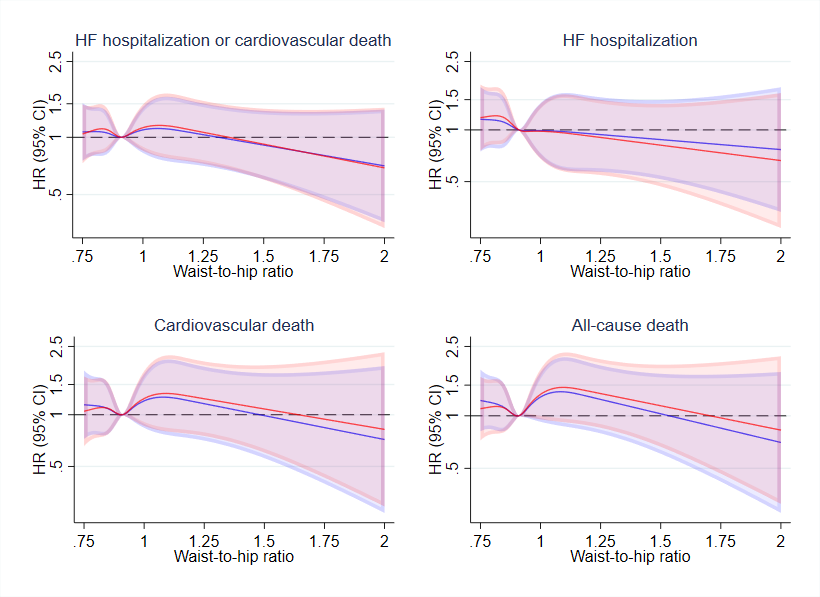


*This figure shows the risk of heart failure hospitalization or cardiovascular death, its components, and all-cause death, according to continuous waist-to-hip ratio. The solid line represents the hazard ratio and the shaded area the 95% CI. The reference is the median waist-to-hip ratio.*

*The blue spline is adjusted for treatment and region. The red spline is adjusted for treatment, age, region, systolic blood pressure, heart rate, estimated glomerular filtration rate, left ventricular ejection fraction, log of N-terminal pro-B-type natriuretic peptide, body mass index, New York Heart Association functional class, heart failure etiology, duration of heart failure, prior heart failure hospitalization, a history of diabetes, and atrial fibrillation.*

*CI, confidence interval; HF, heart failure; HR, hazard ratio.*
